# The Systematic Medical Appraisal, Referral and Treatment (SMART) Mental Health Project: Development and Testing of Electronic Decision Support System and Formative Research to Understand Perceptions about Mental Health in Rural India

**DOI:** 10.1371/journal.pone.0164404

**Published:** 2016-10-12

**Authors:** Pallab K Maulik, Abha Tewari, Siddhardha Devarapalli, Sudha Kallakuri, Anushka Patel

**Affiliations:** 1 The George Institute for Global Health, New Delhi, India; 2 The George Institute for Global Health, Oxford University, Oxford, United Kingdom; 3 The George Institute for Global Health, Sydney, Australia; 4 Department of Medicine, University of Sydney, Sydney, Australia; Médecins Sans Frontières (MSF), INDIA

## Abstract

**Introduction:**

Common mental disorders (CMD) such as depression, suicidal risk and emotional/medically unexplained complaints affect a large number of people in India, but few receive appropriate care. Key reasons for this include few trained mental health professionals and stigma associated with mental health. A potential approach to address poor access to care is by training village healthcare workers in providing basic mental health care, and harnessing India’s vast mobile network to support such workers using mobile-based applications. We propose an intervention to implement such an approach that incorporates the use of mobile-based electronic decision support systems (EDSS) to provide mental health services for CMD, combined with a community-based anti-stigma campaign. This will be implemented and evaluated across 42 villages in Andhra Pradesh, a south Indian state. This paper discusses the development and testing of the EDSS, and the formative research that informed the anti-stigma campaign.

**Materials and Methods:**

The development of the EDSS used an iterative process that was validated against clinical diagnosis. A mixed methods approach tested the user acceptability of the EDSS. Focus group discussions and in-depth interviews provided community-level perceptions about mental health. This study involved 3 villages and one primary health centre.

**Results:**

The EDSS application was found to be acceptable, but some modifications were needed. The community lacked adequate knowledge about CMD and its treatment and there was stigma associated with mental illness. Faith and traditional healers were considered to be important mental health service providers.

**Discussion:**

A number of barriers and facilitators were identified in implementing the intervention analysed in a framework using Andersen’s behavioural model of health services use.

**Conclusion:**

The findings assisted with refining the intervention prior to large-scale implementation and evaluation.

## Introduction

Mental disorders including substance use disorders and self-harm account for 8.5% of all Disability Adjusted Life-years (DALYs) worldwide [1]. Data from community-based studies showed that between 13–50% of people suffer from common mental disorders such as depression, anxiety disorder, and somatic complaints [2]. A systematic review reported that in India the burden of mental and behavioral disorders in the community ranged from 9.5 to 103 per 1000 population [3]. Differences in case definitions and methods of data collection accounted for most of the wide variation in estimates. Though treatment for many mental disorders is now available, a large proportion of people with these conditions do not receive adequate care in low and middle income countries (LMIC), such as India. The treatment gap—the gap between the proportion of people suffering from mental disorders and the proportion of affected individuals accessing adequate mental health services—in LMIC, is close to 75–85% [4]. In India, the gap is more in rural regions. The reasons for treatment gap are multi-factorial, and include poor awareness and stigma about mental disorders and discrimination related to mental health services use; lack of trained mental health professionals to provide care; lack of adequate infrastructure and treatment facilities for mental disorders; and absence of adequate political support to develop resources to provide community-based mental health services [5–7].

One potential approach to address this treatment gap is by training rural primary care workers in basic mental health care and empowering them with suitable tools to provide such care. The objective would be to shift some of the burden of providing basic mental health services from trained mental health professionals to primary care health workers who are able to take on such responsibilities after training, thus offsetting one of the system level barriers to mental health care, which is lack of trained mental health professionals. Task shifting and training primary care workers in providing care for a number of health disciplines including mental health have been used in many countries, but the evidence on effectiveness remains sparse [8]. Developing scalable and sustainable models are a challenge that could be addressed by harnessing the enormous potential of mobile technology, especially in areas where physical distances can be a barrier to accessing expert mental health services. With this in mind, we have developed and are evaluating a mobile technology based intervention in rural Andhra Pradesh, a Southern state of India. The overall objectives are to develop and evaluate the feasibility, acceptability and preliminary effectiveness of a multifaceted primary healthcare worker intervention that utilises a mobile technology based electronic decision support system (EDSS) to improve the identification and management of individuals aged ≥18 years with common mental disorders (CMD) such as depression, suicidal intent/self-harm, and other emotional or medically unexplained complaints that are often related to stress. This paper outlines:

1. Development and testing of the EDSS for Accredited Social Health Activists (ASHA) and primary health care (PHC) doctors using mixed methods approach
2. Perceptions of the community towards mental health using qualitative methods.

The context for the study has been reported in detail elsewhere [9]. In brief, the study is based in rural areas where primary health care is provided by health workers based in villages. ASHAs are lay women village health workers and are primarily responsible for providing basic maternal and child care through government funded schemes. They are reimbursed by the schemes using a performance based incentive model that allows them to work independently outside that time. Each ASHA caters to the health needs of about 1000 villagers. The doctor in the PHC is responsible for their overall performance. Each PHC caters to a population of 20,000–30,000 from number of villages and is staffed by one doctor and supporting medical staff. The PHC doctor, supporting paramedical and nursing staff, and ASHAs, form the core of the health delivery system across rural India. Patients from the PHC can be referred to the next level of care either at rural hospitals or district hospitals. The district hospitals have trained mental health professionals including psychiatrists. Ethics approval for this study was obtained from the Independent Ethics Committee of the Centre for Chronic Disease Control, New Delhi. All participants provided informed written consent and the study followed ethical guidelines laid down by the Declaration of Helsinki.

## Materials and Methods

Development and validation of the EDSS was done between March-December 2014. The field testing of EDSS, and the qualitative research to understand the communities’ perception about mental health was conducted between January-March 2015.

### Study site

The study was conducted in villages in West Godavari District of Andhra Pradesh. A PHC was identified based on distance from the field office, and prior research experience working with that PHC. It was ensured that this particular PHC was not part of any other active study and was not one of the PHCs planned for inclusion in the main study. The villages were selected using purposive sampling, after accounting for population size, availability of ASHAs, and distance from the PHC. The aim was to select villages which were not too big (population <2000), had appropriate number of ASHAs and was within easy commute of the PHC to enable any patient to travel to the PHC for treatment. Six villages within easy commute of the PHC were identified but only 3 villages met all criteria. The village leaders and administrative bodies were informed about the purpose of the study, and prior permission was sought from them. Qualitative research to understand the communities’ perceptions about mental health was conducted in two out of the three villages prior to testing the EDSS.

### Development and field testing of EDSS for ASHAs and PHC doctors

**Fig 1:**
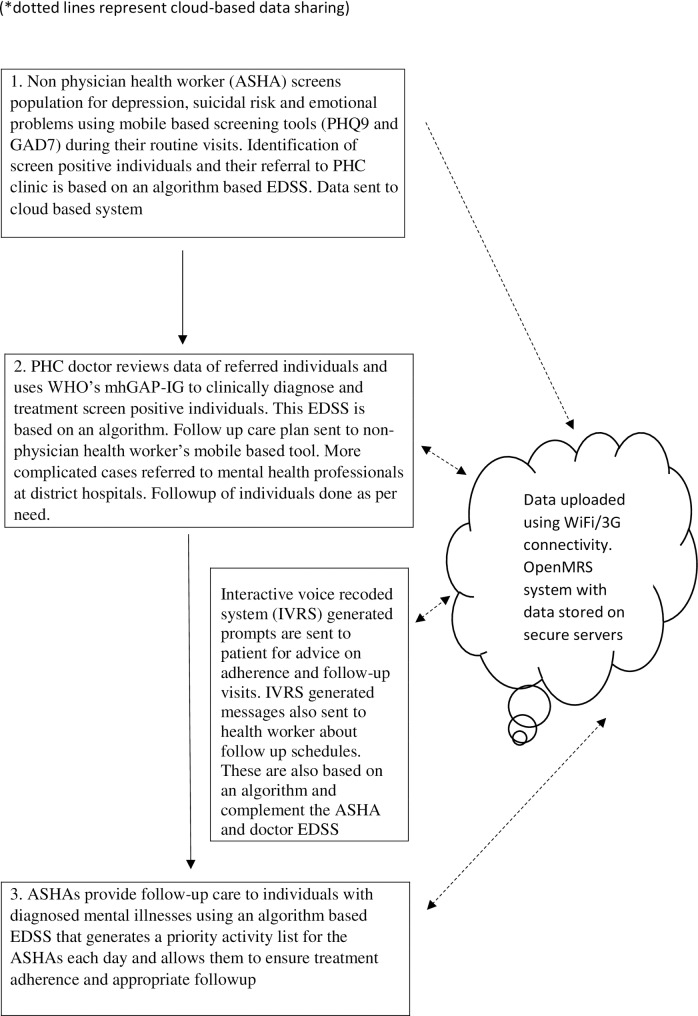
Electronic decision support tools used at different stages of the intervention.

Two separate EDSS were developed–one for ASHAs and another for PHC doctors–and used at three different stages of the intervention (Fig 1). The EDSS software was developed on an Android platform, optimised for 7 inch tablets. The EDSS for ASHAs was a screening tool based on the Patient Health Questionnaire-9 Item (PHQ9) [10, 11] and Generalized Anxiety Disorder-7 Item (GAD7) instruments [11, 12]. Since anxiety and depression often occur simultaneously, those with a score ≥10 on either scale or who responded positively to the question enquiring about suicidal intent on the PHQ9 were considered as screen positive and were immediately referred to the PHC. Both these scales had been validated in India and translated into Telugu, the local language [13].

The diagnostic and management guideline used by the PHC doctor was the Mental Health Gap Action Programme–Intervention guide (mhGAP-IG) [14] and focused on three conditions—*depression*, *suicidal intent/self- harm*, *and other emotional or medically unexplained complaints*. The mhGAP- IG was in English. The algorithm needed to be modified based on multiple iterations and feedback from the research team and a psychiatrist. The sequence of questions and the algorithm was streamlined such that the same question was not repeated in situations where an individual had comorbid mental disorders, eg., depression with psychotic features and alcohol use disorder, and at the end of the assessment one could identify both the primary as well as comorbid mental health conditions. This also allowed the doctor to make a more informed decision about the treatment recommendations provided by the EDSS. In general, these recommendations targeted treatment of simpler cases of depression and suicide risk by the PHC doctor, while referral to mental health specialists, available at the nearest district hospital, was emphasized for more complex problems. The treatment algorithm provided guidelines about both pharmacological and psychological treatment, and indicated when a referral was needed.

#### Validation of the EDSS for ASHAs

More than 50 different profiles were entered in the ASHA EDSS and the ability of the EDSS to identify screen positive cases correctly based on the algorithm developed for this study, was matched with the paper-based PHQ9 and GAD7 tools. A perfect match was obtained.

#### Validation of the EDSS for PHC doctors

Once developed the tool was populated with 20 mock patient profiles by another researcher and the diagnoses made by it was compared against the original mhGAP-IG independently by a psychiatrist, for validation. There was perfect agreement between the mobile based EDSS and the paper based mhGAP-IG guidelines. This version was tested in the field.

#### Field testing and evaluation of the EDSS using mixed methods

Four ASHAs and one PHC doctor from the PHC associated with the three villages chosen for this study were included. Both the ASHAs and the doctor were initially trained about mental health symptoms and its management, and the EDSS that they would use. Besides training on pharmacotherapy, the doctor was also provided training on simple psychological therapies that involved talking to the individual about stress, treatment adherence, social support, and need for engagement in activities. The ASHAs were trained for one week using a training manual and the doctors were provided an intensive 2 hour training and presentation using the mhGAP-IG tool. Before handing over the EDSS, both ASHAs and doctor were provided additional booster training on how to navigate the tools and all their doubts were clarified. This occurred about 2–3 weeks later.

Each ASHA was asked to screen 20 individuals and refer those who screened positive to the PHC doctor. They were advised to screen in an opportunistic manner and combine it with their routine home visits. Additionally, they were also advised to use their spare time whenever possible. The PHC doctor used the mobile based mhGAP-IG application to diagnose referred individuals and provide appropriate treatment. Screen positive individuals visited the PHC outpatient clinic on a particular day and time of the week as indicated by the doctor. This allowed the doctor to devote more time to these patients, without affecting the routine clinical commitments. The work of both the ASHAs and the doctor were monitored by the research staff, and their time was financially compensated at the rates provided by the government.

Quantitative data was collected by using a mobile–based questionnaire used by ASHAs that also had PHQ9 and GAD7 [10, 12] embedded within it. Data about service usage was tracked by gathering such information from the field by following up on individuals who had been screened positive and were asked to visit the PHC doctor and supplementing it with information gathered from the data obtained through the EDSS. The questionnaire provided details about the population screened, e.g., age, gender, depression and anxiety symptoms, total depression and anxiety scores, whether a person qualified as screen positive for CMD. Analytics from the EDSS allowed us to identify the number of individuals screened by each ASHA, number of screenings done on a particular day by a particular ASHA (which helped in monitoring the activities of ASHAs), number of screen positive individuals e who visited the PHC doctor, final confirmed diagnosis as per the doctor’s EDSS, and the appropriateness of the treatment provided.

Qualitative data provided additional information about the usability of the applications (S1 Table). Open ended questions with additional probes to clarify any particular issues were used. One focus group discussion (FGD) was done for the ASHAs and a semi-structured interview was conducted with the doctor. Information was obtained about their experiences in using the EDSS, including facilitators and barriers during its implementation. Suggestions for improvement of the EDSS per se, or specific aspects related to the processes involved in implementing the intervention in the community were also enquired. Additionally, field notes and observations made by field investigators were used to supplement the information.

### Perceptions of the community towards mental health

#### Focus Group Discussions (FGD) and in-depth interviews (IDI)

This part of the research mainly focused on gathering information about the knowledge and attitude of the community, ASHAs, and the PHC doctor about mental health and mental health services. It was conducted in 2 villages.

Altogether four FGDs were conducted- two with male and one with female members of the community and one FGD with the ASHAs. An IDI was conducted with the PHC doctor. Purposive sampling was used for the recruitment of participants from the community to ensure participation of both genders, as women generally avoid attending such meetings in villages.

Two team members—one moderator and one note-taker—conducted the FGDs and IDI. The moderator explained the purpose and procedure of the FGDs and IDI to the participants, and the note-taker was responsible for audio-taping the discussions and taking notes during interviews.

The FGDs and IDI were conducted in the local language, Telugu, at a scheduled time and place, as per the convenience of the participants and at a venue within the community. The confidentiality of responses was assured.

The FGDs and IDI were done using interview guidelines which comprised of open-ended questions and prompts. The questions were translated from English to Telugu. The questions were asked to elicit participants’ responses about knowledge related to mental disorders and their treatment; about their practices or beliefs related to culturally relevant methods to treat mental disorders; about their opinion on social inclusion of people with mental disorders and suggestions about how to increase mental health knowledge and reduce stigma against mental illness in the community.

The IDI involving the doctor focused on different kinds of mental disorders seen by the doctor in the community, stigma, types of treatment available, use of traditional faith healers as mental health service providers by the community, importance of increasing mental health awareness in the community and best way to deliver mental health care in the community.

#### Data management and analyses

All quantitative data from the EDSS were automatically uploaded into secure electronic data bases from where de-identified data was downloaded to conduct the analyses. Descriptive analyses was undertaken to provide an understanding about the ability of both the ASHA and doctor EDSS to identify screen positive individuals and diagnose them clinically. Some socio-demographic correlates were also analyzed. STATA 13 [15] was used for the analyses.

The qualitative interviews were digitally recorded. The tapes were first transcribed to Telugu and then translated to English. The research team familiarized with the response by going through the data repeatedly. During the familiarization process, broad thematic areas were identified and a coding scheme was formulated using an inductive approach. All transcripts were reviewed by three researchers (SK, AT, SD) to identify recurrent themes across individuals and groups, which were then refined into codes, using an inductive method. Next, two researchers (SK, AT), working together, defined each code category and then individually proceeded to code the text of the interviews and FGD data. This ensured reliability and reduced bias. Discrepancies in coding were discussed and a consensus was reached. Interviews and focus groups were analyzed separately using NVIVO 9. Thematic analysis based on an analytic framework [16, 17] was used. No systematic difference was observed among the FGDs and interviews so the data was pooled together for analysis.

## Results

The results of the development and testing of the EDSS and qualitative research to understand the perceptions about mental health in the community are described below.

### Mixed method evaluation of the EDSS for ASHAs and PHC doctors

#### Quantitative analysis

A total of 77 community members were screened by ASHAs (Male– 50.7%, Mean age (SD)– 35.2 years (12.7), Range 18–72 years). Twelve (15.6%) were found to be screen positive and were referred to the doctor, 7 of whom were women. Compared to the age of those who did not need a referral to doctor (Mean 33.3 years, SD 1.3 years), those who needed referral, were significantly older (Mean 45.9 years, SD 5.0 years, p < .001). Nineteen (24.7%) had scored ≥5 on either the PHQ9/GAD7, suggestive of depression. Moderate to severe depression (≥10 on PHQ9/GAD7) was identified in 11.7% of the screened population. Five out of the twelve persons who were referred to the doctor actually visited the doctor. One of them was found to have depression with psychotic features and was referred to a psychiatrist.

#### Qualitative analyses

During the FGD, the ASHAs mentioned that they felt empowered to talk to the community members about mental health with the new skills. They reported that the community also was more willing to discuss their mental health symptoms with them as they were asking more mental health specific questions. They were satisfied with the training and support provided by the institute. They mentioned that initially they required more support from the research staff in using the tool, but later they were comfortable.

They cited that the two main reasons for people not visiting the doctor were distance of the PHC from their homes, and old age which prevented them from traveling long distances. Additionally they felt that it would be difficult to do the intervention effectively during harvest season as both ASHAs and many villagers work as seasonal labourers in the fields during harvest. Overall, they felt that the project would have definite benefits for the community.

During the semi-structured interview with the PHC doctor, no major issues with the EDSS were identified. The doctor felt that the training received was useful and had helped him to use the tool quite effectively. However, he mentioned that the length of the mhGAP-IG was too long and that may be an issue in the clinic. He also observed that many elderly people complain of ‘a wish to be dead’–a culturally relevant style of expressing their frustrations related to their physical or social problems, due to age-related factors, which is not a true reflection of the severity of their depressed mood. Hence a low threshold for identifying suicidal risk may lead to increased number of false positive cases. The role played by the research team to coordinate the referral of screen-positive individuals to the PHC doctor was also mentioned. The need to ensure some system to facilitate continuity of care between ASHAs and PHC doctors was stressed by the doctor. Absence of medications, like anti-depressants, to treat people with CMD, hence the need to refer all such cases to the next level of care, was identified as a potential barrier to accessing care at PHCs by the doctor. The need to update the EDSS application regularly was also indicated as a potential challenge to deliver care smoothly.

Additionally field notes written by the field investigators also corroborated some of the points by indicated by ASHAs. They also mentioned that it was difficult to motivate people to go to see the doctor due to distances, and lack of time, as often their regular farm-related work overlapped the timing of the PHC clinic. Given many of them were daily wage earners, missing work was not an option for most, especially the men. The notes also indicated that the field investigators had to play a crucial role to ensure that screen positive individuals visit the PHC doctor by acting as facilitators in the whole process of linking the individual with the doctor.

#### Community-based qualitative research to gather information about perceptions of the community towards mental health

A total of 25 community members (17 male; 8 female; age 20–70 years) and 6 ASHAs (females aged 35–46 years) participated in the FGDs. The participants were illiterate or had primary and secondary school level education. Most of the male participants worked as farmers or labourers working in the fields with additional jobs as small storekeepers, or other small businesses. A few had government jobs. Most of the female participants were housewives or engaged as teachers or health workers. In almost all the discussion the village heads were also present.

Each FGD took approximately 45 minutes and the IDI lasted for 30–35 minutes. The results of both the FGDs and IDI are presented below under major themes, and a summary is presented under S2 Table.

#### 1. Perception about mental health and mental disorders

In general, the community members perceived that people with mental disorder are not able to do their regular activities properly. According to them mental disorders are due to personal or situational problems. Some participants felt that any kind of stress, pressure or tension leads to mental disorders, whereas others felt that this is due to loss of immediate family members or close friends. One of the participants indicated, *“There are many people with tension due to loss in business or somebody’s death in the family”* (35 years old male participant).

Some participants thought that financial crisis or loss in business are the cause of mental disorders. One female participant was particularly concerned about pregnant women. According to her, pregnant women get anxious because of the fear of childbirth or procedures to be adopted for delivery, such as Caesarian section or normal delivery, and this worry leads to symptoms of mental disorders.

Most participants remarked on the connection between mental health problems and excessive consumption of alcohol. Most people believed that excessive drinking and drunkenness were associated with interpersonal quarrels and domestic violence. One participant mentioned, “*Alcohol consumption is the root cause for mental disorder and all other problems”* (45 years old male participant).

ASHA’s opined that often young married women suffer humiliation due to arguments /violence with husband and mother-in-law, which leads to mental disorders—*“Young married women suffer from problems in marital relations resulting in mental disorder”* (34 years old ASHA).

Few respondents were able to describe someone who had mental disorder. One community member said, “W*e do not observe anyone particularly as we are engaged in our daily work*.*”* (72 years old male participant).

Most of the participants felt that mental disorder was a major health problem and it ruined many lives. Some participants who were able to indicate the signs/ symptoms in detail. They felt that people with mental disorder want to be left alone.

Some other participants mentioned that people considered mental disorder as a form of paranormal or supernatural form of activity. One participant said, *“By seeing these symptoms our people think that it is the effect of ghost or some spirit”* (48 years old female participant).

The IDI with the doctor identified daily stress, economic hassles, and loneliness amongst the elderly as some of the causative factors for mental disorders. The doctor was able to differentiate between CMD and severe mental disorders.

#### 2. Lack of awareness about treatment facilities

Service related issues were mentioned by several participants, notably within the context of a desire to seek treatment in the community settings. Most of the participants were not aware about the treatment options and indicated that no medicines are available for people suffering from mental disorders. One participant indicated, *“There is medicine for all illness except mental disorder”* (52 years old male participant).

A majority of the participants felt that services are not available locally and it is difficult to consult a psychiatrist at the district level hospital—*“In our locality there are no facilities for mental health problem*, *we have only one PHC where they treat fever and other physical problem”* (43 years old male participant).

#### 3. Community beliefs and practices

Faith healers and traditional health practitioners were generally well accepted and their roles appreciated by the community people. The majority of the participants thought that irrespective of religion everyone believes in traditional healers /faith healers for treatment of mental disorders. ASHA’s mentioned that if any person suffers from a mental disorder, the first step is to take the person to a faith healer where they are given amulets /thread and other various items, and only if there is no improvement will visiting a doctor or hospital for treatment be considered.

During the IDI, the doctor mentioned that villagers think that traditional healers play an important role in treating people with mental disorders due to cultural beliefs and often such is a shared practice based on the experience of one person who had used such services.

#### 4. Attitude towards people with mental disorders

Most of the participants shared the view that people were not concerned about persons with mental disorders. An adult male participant said, *“We are not interested in observing people with mental health problems*” (65 years old male participant).They also felt that often they are looked down upon or abused, harmed physically and neglected, even by family members and relatives. Often people make jokes and tease them. A respondent mentioned, “*the problem is usually people don’t help who need such help; in fact they will ridicule and throw stones at such people but genuinely nobody will help people”* (45 years old male participant). Most of the time they are neglected by the family and friends. There were few participants who felt that if a person suffered from mental disorders, their relatives were more interested about usurping the property of that person rather than helping the ill person.

The doctor identified stigma and lack of awareness about mental health in the community as a major issue.

#### 5. Suggestions from the participants

All participants unanimously suggested creating awareness about mental disorders among community members, so that people suffering from such disorders can get support and treatment in time. One participant suggested forming a committee where female members can easily reach out and register their complaints for domestic violence, for example, if it was the causal factor for mental health problems.

Both community members and ASHAs suggested various methods to create awareness, including creating social networks, and group meetings to educate community members. They also suggested that doctors from government hospitals, celebrities, and other educated people should be involved in the awareness campaign as people in the community respect such persons, especially if it includes a doctor. Another suggestion was to organize door-to-door campaigns for those who cannot share their problem in public and need personal attention. They felt that all such meetings should be organized in each locality so that more people from across the community can attend them and gain knowledge. An important feedback was not to conduct the meetings near the village administrative (panchayat) office, as it did not provide enough privacy for everyone to express their opinions due to differences in socio-economic status, further the distances of such offices from their homes can prevent people from attending.

The doctor suggested that the mental health awareness campaigns are needed and should involve volunteers, religious persons, teachers, local leaders and health professionals from local clinics.

## Discussion

The knowledge gained while developing and testing the EDSS and obtaining communities’ perception about mental health, were critical to the study.

### Development of the EDSS for ASHA and PHC doctors

The iterative process of refining the applications helped to streamline the algorithm and make them more user-friendly. The quantitative phase provided some preliminary information about the ability of the tool to identify CMD in the community and provided us with an understanding of the usability of the application. Overall, both the ASHA and doctor EDSS performed as desired and was able to identify people with CMD, and provide diagnosis and treatment options to them. Based on the experience further modifications were made to the EDSS and the processes for the intervention.

Overall, a number of strategies included in the study could be mapped onto Andersen’s modified Behavioural Model of Health Services Use [18] (Table 1). Based on literature and research experience some potential barriers were identified prior to the formative research and strategies were implemented to facilitate mental health services use, e.g, the development of the EDSS system. Other barriers were identified and addressed subsequent to the formative research based on the research findings, e.g., organizing health camps in villages to facilitate easier access to the PHC doctor, developing an algorithm based system by which data is shared between ASHAs and PHC doctors to facilitate followup. The overall outcome of those strategies was to improve the mental health status of the community and to increase utilization of mental health services by screen positive individuals.

**Table 1:** Strategies used to address barriers and facilitate mental health services use based on Andersen’s modified Behavioural model of Health Services Use.

Key component from Andersen’s model	Barriers/Facilitators	Actions taken to address barriers prior to the formative research or subsequently	Anticipated outcome
*Environmental*			
Healthcare system	1. The PHCs are not oriented towards providing mental health services; 2. The primary health workers–ASHAs and doctors—are not suitably trained in identifying and treating mental disorders	*Action taken prior to the formative research* 3. Using an EDSS to screen and diagnose individuals for CMD 4. Empowering ASHAs to screen people in their community by providing training and remunerating their services 5. Motivating doctors by providing them training and remunerating them based on the services they provide *Action taken subsequent to the formative research* 6. Developing a collaborative network with the government to enable the government staff to work on the project **7.** Seeking government support to ensure availability of free anti-depressants in the PHC.	8. The delivery of mental health services at PHC level will be facilitated and evidence based care will be provided
*Population characteristics*			
Predisposing characteristics	9. Beliefs about causality for mental disorders and their treatment was inadequate	*Action taken subsequent to the formative research* **10.** Organizing mental health awareness campaign prior to the intervention.	11. Improvement in health belief and knowledge about mental health will improve leading to increased service use
Enabling resources	12. Key stakeholders needed to be informed about the study and involved to enable better coordination 13. Poor accessibility to PHC due to distances from villages	*Action taken prior to the formative research* 14. Discussions were held with village leaders prior to the formative research and this helped them understand the need for such a project and receive the local administration’s support for the programme. *Action taken subsequent to the formative research* **15.** It was decided that camps would be organized in villages to ensure increased followup with doctors such that villagers need not spend time and money to travel to the PHCs.	16. The enabling factors will facilitate increased uptake of mental health services by streamlining service use
Need	• Perceived need for seeking mental health services was low because of lack of awareness • Primary health workers including PHC doctors were less oriented towards identifying mental disorders	*Action taken prior to the formative research*• The ability of primary health workers including doctors to identify and manage CMD was enhanced by using evidence-based algorithm driven EDSS *Action taken subsequent to the formative research* • Modifying the mental health awareness materials by incorporating some of the culturally relevant issues that were identified during the formative phase helped the community to understand mental health issues better and discuss them more openly	• With increased perceived and evaluative need, identification of CMD and uptake of services will be increased
*Health Behaviour*			
Personal health practises	• The community did not have adequate knowledgeable about mental health symptoms or the need to seek care when stressed or feeling low, or the kind of services available	*Action taken subsequent to the formative research* • Modifying beliefs about mental health and changing perceptions about accessing mental health services through a mental health awareness programme that talks about stress, mental health symptoms and how one may benefit from using one’s social support and accessing mental health services when severe	• It is hoped that with increased awareness will come increased use of mental health services when in need
Use of health services	• The followup of screen positive individuals was a major problem and was affecting service utilization • Low motivation for the screen positive individuals to visit the PHC doctor	*Action taken subsequent to the formative research* • The EDSS was seen as facilitating more efficient care *Action taken subsequent to the formative research* • Developing a system to share information between the ASHA and doctors such that continuity of care and treatment adherence could be ensured more effectively • Developing an IVR system to complement the EDSS to inform ASHAs, doctors and community members about mental health services and treatment that needs to be followed • The doctors were trained to not only use the EDSS but first to use their clinical skills in elucidating symptoms. This would reduce time taken to screen each individual. This was suggested as an alternative to asking each question in sequential order by reading them out from the application.	• These strategies will be able to streamline followup of screen positive individuals and ensure better treatment adherence • The IVRS will complement the system of providing care by providing an additional mechanism to enhance care

### Communities’ perception about mental health

The feedback we received through the FGDs and IDI helped us gather local information about how the community, including health workers, perceive mental health. Information from prior research provided similar themes too and helped us to develop materials for the anti-stigma campaign [19, 20]. The need of a mental health awareness campaign was deemed useful prior to the launch of the intervention, to not only prime the population about the programme and make them more knowledgeable about CMD, but also to reduce stigma against mental health and mental health services. It provided important data that influenced the content of the anti-stigma campaign. We used learnings from this process to modify the language of the campaign materials and place greater emphasis on the issues highlighted by the community, such as, use of traditional healers, poor knowledge about symptoms of CMD and effective treatment, and information about evidence-based mental health services that can be effective in treating people suffering from mental disorders. While integrating mental health services within the routine health services is itself less stigmatizing, the formative research highlighted the need to specifically focus on mental health knowledge, attitude and behavior. This research helped to inform the development of the anti-stigma campaign and modify strategies for delivering the mental health awareness materials.

It highlighted that majority of participants in this study were aware of mental health and some of the symptoms of mental disorders, but their knowledge was limited. They knew little about the potential contribution of modern medicine in treating people with mental disorders and had almost no experience with using mental health services. It was clear that the lack of knowledge was also a cause for stigma against mental health. These findings are in line with many studies which have demonstrated that the ignorance and lack of awareness about mental health are key elements that lead to stigma against mental health and persons with mental disorders [6]. While alcohol was identified as an issue in the community and the need to develop an intervention in future on alcohol use disorders was noted, the current project does not have the scope to include alcohol use disorder.

The majority of the participants shared the view that irrespective of religion everyone believed in the ability of traditional healers /faith healers in treating mental disorders and it was a potential barrier to receiving evidence based care in the PHC. This was similar to previous research that has shown that culturally determined beliefs act as a major barrier in seeking professional help for psychological problems [21].

Most study participants suggested creating awareness about mental disorders among community members so that people suffering from mental illness get support and treatment in time. Studies on creating awareness in developed countries show that community-based programs, with involvement of local communities were effective, and emphasized on public education to increase mental health awareness and thereby influencing help seeking behavior [22].

### Limitations

While the study provided valuable information for the main study, it had its limitations. While a needs assessment was not done prior to the project, a detailed review of literature and evidence from prior studies conducted in the same geographical area as in this project, suggested that depression and suicide were important problems in the community [23]. Additionally the qualitative research highlighted the perceptions of the community about CMD and the need for services for managing CMD and supported the rationale for targeting CMD. The purpose of this study was to test the usability of the EDSS and gather some ideas about the overall perceptions about mental health in the community. The sample size was not powered for any specific mental health outcome. The qualitative analyses used an analytic framework based around thematic analyses. The information obtained by interviewing the community members, all ASHAs and the doctor were quite similar, so while we did not reach thematic saturation for the FGDs with community members per se due to the limited scope of the study and the paucity of time and resources, data collated from all the interviews had achieved thematic saturation. Using strategies recommended earlier [24], the information gathered from the interviews was triangulated against prior research, researchers’ experience working in similar cultural settings, and field notes, and found to have similar themes, increasing validity of the results. The ASHAs and the doctors who are also part of the community, expressed similar themes as identified by the community members. Based on these factors, we are confident that all major themes were identified.

## Conclusion

The study provided an understanding of the key beliefs about mental health prevalent in the community and allowed determination of user acceptability of the EDSS. The results informed further modification of the EDSS and also helped us modify some of our overall research strategies from a behavioural perspective for mental health services use. The information obtained from the community about their perceptions about mental health provided local context to the information that we already had from our prior literature review and helped us fine tune the mental health awareness and anti-stigma campaign further. The formative phase also showed that the EDSS based system of care could be implemented by primary care health workers. While we do acknowledge that the current workforce may be insufficient to suitably scale up this project and similar others, simultaneously across the country, the government is planning to increase the number of non-physician health workers and is using mobile based tools, too. Given such developments it is crucial to understand how primary care health workers use the EDSS for managing CMD, using the knowledge gained through more robust study designs that will lead to development of a cost-effective and scalable program for the country.

## Supporting Information

S1 Dataset Quantitative data from ASHA screening. (XLS)

S1 Table Guidelines for Focus Group Discussions with Community members and ASHAs and Interview with Doctor. (DOCX)

S2 Table Summary of qualitative research findings. (DOCX)

S1 Text SMART Mental Health formative research transcripts. (PDF)
