# Supplementary material for: The Systematic Medical Appraisal, Referral and Treatment (SMART) Mental Health Project: Development and Testing of Electronic Decision Support System and Formative Research to Understand Perceptions about Mental Health in Rural India
Source: PLoS One. 2016 Oct 12;11(10):e0164404. doi: 10.1371/journal.pone.0164404 (PMC5061375; doi:10.1371/journal.pone.0164404)
Supplement: S1 Table — (DOCX) [file pone.0164404.s002.docx]

**S1 Table: Guidelines for Focus Group Discussions with Community members and ASHAs and Interview with Doctor**

| **Guidelines for focus group discussions with Community members** |
| --- |
| - Please tell us how do you identify people experiencing mental disorder in your area? From whom do you usually hear or find out *them? Probe: community workers, Doctors, ASHA’s, friends, relative or any other.* - Generally, how do other community members behave with them? *Probe: behavior of health workers, doctors, friends, relative.* - Do you agree that some kind of stigma is attached to the people with mental disorders in your area? Give details. *Probe: types of stigma, types of discrimination* - What are the different practices that people adopt to tackle such issue? In your opinion, what are the benefits of these practices? Are there harmful effects from these practices? If so, what? [*Probe: benefits, barriers*] - Are you aware of any existing treatment services available in your area? If yes, provide details. If no, what steps should be taken in your community to help people suffering from mental disorder. - Do you think people in your area need some information to bring awareness in the communitiy’s outlook towards mental disorder & its treatment? In your opinion who should be involved to provide such information. *Probe: NGOs, Government, Community leaders, Religious leaders, Teachers? And who else?* - How could we package this information into a programme to deliver to community people/villagers like you? A handout? A talk? Folk music? Street play, rallies? There are many, many ways this can be done- let’s hear your creative ideas for this? - Where this programme should be conducted? In community? At home? And /or where else? Please give details. |
| **Guidelines for focus group discussions with ASHAs** |
| - Please tell us what affects mental health?  *Probe: Biological factors, Any events, Social factors* - What are the common beliefs about causes of mental disorders? Please share your experiences? *Probe: myths, visiting faith healers, cause because of God’s wishes.* - Do you agree that some kind of stigma is attached to the people with mental disorders in your area? Give details. *Probe: types of stigma, types of discrimination* - According to you what are the different ways which we can adopt to reduce stigma in the community? - Do you think mental disorders are curable? If Yes, what are the different ways to treat these disorders? If No, why do you think they are not curable? - As a healthcare worker what initiative you would take if you identify a person with a mental disorder in your community? - Are you aware of some of the common mental disorders? What do you think will happen if common mental disorders are not treated? - According to you do you think mental health services are easily accessible to the community? If yes, provide details. If no, what steps should be taken in your community to increase the accessibility to people suffering from mental disorders? - Do you think people in your area need some information to bring awareness in the community’s outlook towards mental disorder & its treatment? In your opinion who should be involved to provide such information. *Probe: NGOs, Government, Community leaders, Religious leaders, Teachers? And who else?* - Where according to you where and how would be the best place to conduct these community awareness programmes? In community? At home? And /or where else? Pl gives detail. |
| **Guidelines for Interview with Doctor** |
| - Can you please tell us what fractions of the population that you see in your clinic suffer from mental disorders? Probe: what fraction suffer from CMD and SMD? What fraction suffer from depression/stress? What fraction suffer from alcohol use disorders? - Do you feel comfortable treating patients with mental disorders in your practice? Are there any major challenges that you face? - What are the common beliefs about causes of mental disorders within the community? Please share your experiences? Probe*: myths, visiting faith healers, cause because of God’s wishes.* - Do you experience any stigma in the community about mental disorders? Give details. *Probe: types of stigma, types of discrimination* - According to you what is the best way to reduce stigma in the community? *Probe: As healthcare professionals what initiatives need to be taken* - Do you feel there is any stigma among health workers about mental disorders? Give details. *Probe: types of stigma, types of discrimination* - According to you what are the different ways which we can adopt to reduce stigma among health workers? *Probe: As healthcare professionals what initiatives need to be taken?* - How important according to you is to treat common mental disorders? - According to you do you think mental health services are easily accessible and affordable to the community? If yes, provide details. If no, what steps should be taken in your community to increase the accessibility to people suffering from mental disorders? - Do you think people in your area need some information to bring awareness in the communitiy’s outlook towards mental disorder & its treatment? In your opinion who should be involved to provide such information. *Probe: NGOs, Government, Community leaders, Religious leaders, Teachers? And who else?* - Where according to you where and how would be the best place to conduct these community awareness programmes? In community? At home? And /or where else? Pl gives detail. - Any other suggestions? |
