# Supplementary material for: The Systematic Medical Appraisal, Referral and Treatment (SMART) Mental Health Project: Development and Testing of Electronic Decision Support System and Formative Research to Understand Perceptions about Mental Health in Rural India
Source: PLoS One. 2016 Oct 12;11(10):e0164404. doi: 10.1371/journal.pone.0164404 (PMC5061375; doi:10.1371/journal.pone.0164404)
Supplement: S2 Table — (DOCX) [file pone.0164404.s003.docx]

**S2 Table: Summary of qualitative research findings**

| **Themes** | **Key findings** | **Reflective Quotes** |
| --- | --- | --- |
| Perception about mental health and mental disorders | - People with mental disorder are not able to do their regular activities properly. - Different reasons cited for mental disorder were stress, domestic quarrels and abuse, loss of immediate family members/friends and financial crisis, alcohol consumption - Mental disorder was thought of as a form of paranormal or supernatural form of activity | *“There are many people with tension due to loss in business or somebody’s death in the family”* (35 years old male participant)  *“Young married women suffer from problems in marital relations resulting in mental disorder”* (34 years old ASHA)  *“By seeing these symptoms our people think that it is the effect of ghost or some spirit”* (48 years old female participant)  “*Alcohol consumption is the root cause for mental disorder and all other problems”* (45 years old male participant) |
| Lack of awareness about treatment facilities | - Treatment /Services were not available locally - Participants were not aware of treatment facilities | *“There is medicine for all illness except mental disorder”* (52 years old male participant)  *“In our locality there are no facilities for mental health problem, we have only one PHC where they treat fever and other physical problem”* (43 years old male participant) |
| Community beliefs and practices | - Faith healers /traditional healers play an important role in treating people with mental disorders due to cultural beliefs | *“There is a place [traditional healer] nearby to our village. Earlier only 1-2 people used to go there for treatment but now the number has increased to 1000 people”* (56 year old male participant) |
| Attitude towards people with mental disorders | - People were not concerned about persons with mental disorders. - Family members, relatives or neighbors do not behave properly with the persons suffering from mental disorders | *“We are not interested in observing people with mental health problems*” (65 years old male participant)  *“The problem is usually people don’t help who need such help; in fact they will ridicule and throw stones at such people but genuinely nobody will help people”* (45 years old male participant) |
| Suggestions from the participants | - Need to create awareness about mental disorder - Build social networks, and group meetings to educate community members - Organize door-to-door campaigns for those who cannot share their problem in public and need personal attention | *“Organize a big meeting to increase awareness in village…… conduct a meeting on Sundays so that we may come to listen you”* (52 year old female participant)  *“Our message must go to door to door to women and men in a family and they must be given some moral support if they identify any kind of mental tensions it can be cured etc., so these kind of things must be done”* (45 year old male participant) |
