# Supplementary material for: The Systematic Medical Appraisal, Referral and Treatment (SMART) Mental Health Project: Development and Testing of Electronic Decision Support System and Formative Research to Understand Perceptions about Mental Health in Rural India
Source: PLoS One. 2016 Oct 12;11(10):e0164404. doi: 10.1371/journal.pone.0164404 (PMC5061375; doi:10.1371/journal.pone.0164404)
Supplement: S1 Text — (PDF) [file pone.0164404.s004.pdf]

S1 Text: SMART Mental Health formative research transcripts

Focus Group and Interview Data

FGD -1

Date: 21 Jan 2015

Place: Village [Name…..]

Group Details: Males

Moderator: Mr. Siddhardh Devarapalli

Note taker: Sudha Kallakuri

Moderator: A very good morning to everyone. We are very thankful that all of you have come here to discuss about the topic of mental health knowledge and awareness in your village. Before we start I would like to introduce myself. I'm Siddhardh and they are my colleagues Sudha and Balaji. We have come from an organization called George Institute for Global health, Hyderabad. We are here to discuss about mental health awareness in your village. I request you to kindly give your valuable inputs which will be very useful for us to design an awareness programme. We also would request you to speak one by one and keep your mobile phones in silent mode. Please feel free to participate in the discussion and if you have any query please raise your hand, we will try to resolve it. Please speak one after the other for responding to a specific question so that all the answers can be well heard.

M: So Please tell us how do you identify people who are experiencing the mental health problem in your area

P1: We can't face them

I: I mean have you seen anyone… till now

P1: We go to work early in the morning and roam out for some other work so we don't show any interest to observe the people mental health problem.

I: I mean not only outside may be in your home some of your family member may be in tensions and if our dear one dies naturally we will be sad for 2-3 days some people will be sad for a year

and some can't get out of that incidence for years together so this is called as mental health problem so have you seen any person like these

P1: Then there are so many people like that

I: Ok, then tell me about it

P4: like parents are of 50 years age and his son of 25 if the son dies because of HIV or any reason the parent may can't digest it and sometime they are also committing suicide it is also mental health problem. 90 % of the problems are due to such issues.

I: Not only that see some children like students of 10th class or intermediate they get worried about tension some students due to this tension they even don't attend the exam and if they come to know that results may come out they get tensed so students also try to commit suicide So have you seen these kind of people.

P2: No sir

I: They may be adults in the matter of marriage etc., any person who is addicted to alcohol whatever may be the reason; family or financial ….anything.

P5: some of the people drink alcohol to forget their problems rich people may drink for fashion or relaxation but the adults in poor family drink to forget their problems I too drink alcohol I feel good because I forget everything for some time.

P5 and P6: most of them drink to come out of some problem or to forget that problem for some time

I: That was what we call as mental health problem because they are not sharing their problem with others and also not trying to solve their problems they are getting addicted to drugs and alcoholic substances so this is also mental health problem as you said now that this addiction must also be considered as mental health problem that is what we are asking you if we say mental health problem we don't mean by saying mad people who roam on road with torn clothes and all; we are not asking about them we want to know the normal health problem which exists in each and every person, tension is normal to everyone but if a person is suffering with more and continues suffering then it must be considered as mental health problems so as these two people has said about some mental health problem so I request other people too to give some more details regarding this topic.

P2: everyone have problem...

P1: Please tell as you have more experience than us.

P4 and P5 : A friend of mine was died due to financial problem when he was suffering with financial crisis we have given him moral support; and during that time he felt good and dropped

the suicide idea but after 2 years he died. Most of the people don't share their problems with others this will increase the stress or pressure on their mental condition. If we have any other family may support like if I am in any problem my mother or my sister may help me but that may not happen in everyone's family. Some may take small problem as big ones and feel tensed. Recently a boy of 20 years committed suicide by hanging himself; he was suffering from mental health problem. It has been observed that most of them nowadays watch movies are not taking good thing they are practicing the bad which is shown in films when a hero was introduced as drinker or smoker he may change due to some incidence or due to his mother but the audience are not seeing that change they are imitating the hero's behavior if they have seen chiranjeevi (who is a famous hero) smoking cigar they do same if at the end of the film if chiranjeevi has quit smoking than they don't do that they just take inspiration from that cigar part.

I: These smoking and drinking , ideas to commit suicide are nothing but the mental health problems these can be overcome by counseling if we identify the mental health problems in initial stage we can prevent more suicide cases by giving them counseling in the right time a psychologist and many doctors who have done their MBBS can do counseling they also give medicines for some kind of persons who need medicine because sometimes due to some chemical reactions in brain can also cause some kind of thought in a person so to balance them medicines are also compulsory so have you heard about these kind of mental health problems before?

P5: No sir as we are discussing with you, we are now thinking about it otherwise we may not have taken it seriously

I: You sir..

P3: Yes sir as you asked now they are saying if not we will not observe anyone particularly as we are engaged in our work daily

I: So you haven't think about this before or you haven't considered these kind of problems as health problems?

P3: No sir

I: Have you seen any kind of posters regarding this or in any movies? Have you heard about these mental health problems before?

P1: we generally know that pressure and stress may cause health problems

I: Ok can you please explain in detail that when did you hear about these kind of mental health problems

P3: It varies from person to person as it also depends on the atmosphere/situation of their family. First of all any person must be brave to face any kind of problem for example if we have started

a business there will be loss and profit, so we must be prepared for both. If any person faces loss in his business he is depressed and gets thought of committing suicide; they usually think that their death is the solution for that problem.

I: Do you agree with that point?

I: Do you think the person who commits suicide do they think in this way?

P2: Yes there are many

P4: they are so many examples in our village that boy how recently died my friend etc., he decided to die leaving his parents. He was very healthy and his body was similar to that of some body builders in films. He was suffering with some health problems because of which he became very weak. His parents used to worry about him and then he thought he is the reason for their worries and committed suicide. After his death his parents went under depression and seriously fell ill and they became bed ridden.

I: As you are saying in a detailed way I would like to ask you; when did you hear about these kinds of mental health problems?

P3: In business usually there will be up and downs because of which there will be many stresses involved; so many of my friends have given me moral support I faced lot of financial crisis many of my friends gave financial and moral support. I was able to come out of it because of my friends. Similarly so many people used to come and share their problems with me and anybody who used to say that he does not want to live and who has suicide idea if they share that idea with me I would not encourage them I would try to get them out of that idea. Every month I come across at least 2 people. So then I explain to them there is no use if you commit suicide, whatever loss you have incurred in this one month if you work one more hour extra per day you will able to recover that loss in a span on 1year.

I: Along with these kind of suicide thoughts, drinking alcohol is also a mental health problem so as in your village there are ANMs, ASHAs, and many other doctors so have any of them conducted any programmes to treat these kind of mental health problems?

P4: No sir in our country many of us don’t like to work more; see we have 5 ASHAs in our village. If there are any meeting that we conduct and when we invite them, 3 of them will attend other two won’t come. They don’t conduct any kind of programs about all these kind of health problems.

I: Apart from ASHA did anyone come before us to give services for mental or physical health problems?

P2: No, Sir

P4: Due to illiteracy there was no idea about all these things but now a days we can see the change so slowly changes are taking place. When we have seen mineral water then we got an idea to keep a water filter system in our village. So slowly changes are taking place; now at least one member of family is literate. In 1993 I was studying my 10th class then also there were not many in our village who were studying; but things are changing gradually and definitely there is a difference in the village and town atmosphere with the number of people who are being educated.

I: If you come to know that a person is suffering from mental health problem how is he treated in your village? How is he considered?

P4: The problem is usually people don't help who need such help; in fact they will ridicule and throw stones at such people but genuinely nobody will help people. Say for instance if a person became alcoholic or suffers some kind of addiction, generally people think to take off his land and property; if our friend or our neighbor is facing trouble they are very few who think to help them out. I'm telling this out of experience as I have been around with such people since childhood.

I: Ok. Yes, You were saying something?

P5: No

I: What is your name and his name?

P5: My name is Balaji and his name is Vasu; we all are of 10 friends we share our problems, people who give moral support for any problem are also less in these days. As our president rightly pointed even if a person suffers from any kind of financial problem or personal problem there are very few people who say that don't worry everything will be ok.

I: You sir, please share your thoughts….I would like to know how things happen in this village as you belong from here you may know better than me. What you think, have you faced any person who have mental health problem? you…

P6: No…

P6: Nowadays people don't listen to anyone they don't respect the words of elders at least.

P3: You are saying something which is out of this topic

I: No, No you just tell us we are here to listen to you

P6: they don't listen to us if we say any advice and if we warn them about future. The younger generation is not acting properly.

I: Do you think a special treatment is needed for the people who is suffering from these kind of mental health problems?

P6: After listening to you we think it is necessary

I: Before this have you felt it is necessary to see a person suffering from any mental health problem he/she needs a special treatment

P3: There is a proverb that there is medicine for everything but not for mental health so we haven't thought in that way until now. As you are saying that counseling may work better. Conducting programs and meetings may give awareness to people; now we came to know that there is also treatment and medicines for mental health problems.

I: Have you heard this proverb

P1: Yes

I: What do you say? Is it correct?

P1: Yes it is

I: So do you think that by treatment these kind of mental health problems can be solved?

P1: After listening you I came to know that it can be solved by treatment; I was not aware about it earlier

I: You sir…in some places if a person is suffering from mental health problem they take them to special temples or to babas; so does it happen in your village too?

P4& P3: Yes sir, Irrespective of religion everyone believes this Christians, Hindu, Muslims all of them do this. By these kind of beliefs many so called babas are earning lots of money.

I: Is there any special temple/such places in your village where people visit for these kind of problems?

P3: Yes there is a place nearby to our village. Earlier the fee for the people who visited that place was Rs 100 and now it is Rs 500. All this started just 6 months ago. Earlier this just started with 1-2 people who used to go there now the number has reached 1000 people. There is a belief in our a village about a village who finds about any robbery/theft that's has happened by putting some substance in the hands of the people who have lost their goods/valuables.

P4: When an object is lost that itself creates so much of worry and tension in a person's mind. He goes to these kind of baba who take Rs 1000 for those kind of things and if he show any person who stolen that object there will be another worry what will happen if we suspect him is he is the one or not and if we go to police taking this as proof they don't believe it. It is really time consuming and waste of money process and also adds to a lot of tension in a person. These babas

also know that these people are vulnerable and they take advantage of these people's weakness and try to shell out as much money as possible from these people.

P5: You have asked correct question we believe in god and we also think to help needy people but by giving 1000rs to these kind of superstitions is really waste of money. Once a baba came to my house told something and my sister and mother told me to give Rs2000 to him. It was better to feed 100 people with that money rather than giving him. For e.g. It is said that early in the morning if the sunrays fall in bedroom most of the skin diseases can be reduced. So keeping this in mind many people say to place bedroom in this side and that side; some of them say that these are predictions. They just say something and it might happen in our life which may be just a mere co incidence but we believe that he have some kind of power and he can predict our future etc. Recently I told my wife that I have seen a snake she immediately brought an astrologer home he told me to do some things to save my self some people like you at least two people from you must be there in village to change people's way of thinking as they don't listen to us .

I: Can you say anything about these babas and these kind of beliefs.

P5: we believe in god

P5: Some of these elder people don't trust

I: If we are suffering with any kind of mental health problem if we share it problem won't get solved but at least some of its stress reduces. Similarly if we go to temple problems won't get solved but our belief in god gives us strength that the problem will be solved soon it gives peace of mind some of the people go to babas for solution they won't solve problem they are there to grab our money so it is better not to believe them.

P3: babas know that if a person is coming to him then he is in problem so he first says that " you are suffering from lot of problems" then the person feels like the baba knows everything.

I: As you have discussed about these mental health problem so do you think that we have to bring awareness in people?

P3: Yes it is necessary

I: In which way we can bring awareness in people of your village?

P5: Nowadays people are not ready to listen anyone

I: in which way must we convey the message about mental health problems?

P3: BY conducting meetings

P1: At least monthly once meeting must be conducted to educate people about mental health problems

[cross talk]

I: who must convey this message to people?
P3: For the youth as they are more interested in film stars so if a hero says about it they will listen more attentively. As Narendra Modi said to keep our surroundings clean or to use toilets so we are also following it. Similarly a message should be conveyed by such famous personalities.

I: You mean to say that these kind of meetings must be held at least once in a month and the other thing is the message must be conveyed by any kind of popular people like hero's or honest politicians, any more suggestions?

P6: film stars

I: film star and..

P1: Politicians

P2: The message must be conveyed in any kind of big meetings then only it can reach large number of people now as there is lot of media out there news spread all over the world very fast.

I: How would it be if we include ASHA's, and doctor in these kind of programs? As doctors know more than anyone

P3: If they attend their duties well we can include them too. In earlier days there was no awareness of taking polio drops but now our village kids are coming themselves to take polio drops without the help of their parents.

I: You mean awareness can be increased if we conduct any meetings? Where we have to make meeting what must be the venue in any hospital or school?

P3: This is center of the village. If we can do the meeting here everyone can attend especially in the evening time as we go to work. After 4 in the evening everyone comes here so many people can attend.

I: Okay …..

P3: We will co-operate with you no problem.

FGD 2

Date: 21 Jan 2015

Place: Village [Name…..]

Group Details: Females

Moderator: Ms. Sudha Kallakuri

Note taker: Mr. Siddhardh

I: Greetings, My name is is Sudha we are from George institute for global health organization. at we would like to ask you some questions to know how many of you are aware of mental health problems. So your details and opinions are very valuable for us to take any step in future. Ok…so we would like to ask you questions and request you answer r it one by one so that to understand clearly and other thing is please don't hesitate to share your opinions you can talk anything without hesitation because every word that you speak is important for us. Please answer voluntarily, there is nothing to get afraid it is just a discussion where you can tell or share your opinions freely.

I: The main aim of this survey is as I told you before that we are from George institute of global health organization here we identify and try to solve the mental health problems During this survey awareness regarding physical and mental health will be given and all will discuss about the rumors surrounding the mental health problems after some time we will talk about normal mental health problems generally due to family problems , financial problems we can feel the stress, pressure, anxiety, or depression it may be big or small problem and not sharing with any other these kind of symptoms are called as normal mental health problems these kind of people search for drugs or alcohol to forget their problems and after sometime they may also try to commit suicide so these all considered as general health problems so we are going to discuss about this topic so please share your opinions freely with us

I: This is a consent form here you have to sign the form, those who can't sign we can also provide stamp pad to you for thumb impression keep the paper with you we will take it at the end and I am requesting you once again to speak don't sit idle please talk one by one

P1: I have a doubt, Are you researchers? Your thesis will be printed as book and published right?

I: No you are talking about Ph.D. But we are doing job as research fellows so the study is about how are the mental health problem in village atmosphere. By knowing about this we try to involve with the PHCs to help and solve these kind of problem in village. If we ignore these kind of mental health problems it will create lot of problems. Further if it is identified in the initial stage and if right treatment is taken at right time to the right person like it may be doctor, ANM, or ASHA after giving best training to them Dr.harish who is the doctor of your PHC has taken training last week especially about mental health problems we have to know how much are the people aware of mental health problems and must search for the means to create more awareness among people

P1: ok you came here to know about all these things but now you are recording our names too

I: No, We just asked your name for introduction not to record your details.

P1: As it is here that our details and the thing we say will not be revealed to anyone it will be confidential.

I: It means we will relate to what you said by creating a unique ID and we will give Number to you so that number will be your identity we will not pin point any person.

P1: My answers may be different from other I may give different proofs

I: That's want we want we don't want similarities we want to know opinions irrespective of community.

P1: You may get more information with other community

I: we have conducted one discussion with male people in morning we haven't asked them from which community they belong to. We just asked their names and opinions we are not getting into other details your opinions may apply if we are conducting survey with groups.

P1: Yes groups

I: But..

P1: I may withdraw

I: It is up to you as it is written in your consent that you are free to withdraw however each and everyone's opinion will add a lot of quality to the data that we intend to collect and it may help us in creating new ideas to conduct awareness programmes.

I: lets start please move forward we need everyone's opinions

I: Greetings my name is sudha we are from George institute we are here to have a discussion with you on mental health here you can voluntarily express your opinions please don't hesitate to share your opinions please talk one by one and please respect other opinions.

P: All of them together say "Yes let us start"

I: Ok... Please tell us about mental health problem have you heard about this before?

P2: I have heard and seen about it in our family. My son was suffering from mental health problem due to tension about his education and health. My son is taking treatment from 6 months. We have seen good results. During the treatment doctors advised him to continue the treatment for 4-5 months but this is really a major problem we can't ignore it as a small thing. People who don't have much awareness about it may face many problems if it is not identified in

the initial stage and it they don't go for treatment immediately they may face many problem like my son…..(and she broke down)

I: Ok have you seen or heard any person who is suffering from mental health problem

P3: We don't have such problem in our family

I: Do you know anything about mental health before

P4: No madam

I: You madam

P5: I heard about my nephew

I: you haven't heard about it before

P6: No

P1: I am very interested in medical domain I read about diseases and make myself aware of it I heard about her son as they told me about him. I told them that this is not superstition like some bad omen etc. I advised them to take him to hospital as I have read in newspaper about mental health problems. I told them to consider it as a health problem so I advised them to go to doctor and take medications we generally take for physical illness. They followed it and I have seen satya meva jayate programme on television which was hosted by film actor Aamir khan. Everyone must watch it for awareness. Other diseases like T.B and tumors may come due to heredity but I do have some doubt that, is this mental health problems are also hereditary or it is due to some other reason?

I: In the interest of time and as we are in discussion we will clarify your doubt after this program

P4: I have seen her son he was depressed worried and sometimes he cries and also wanted to commit suicide and he usually prefers to remain lonely. So by seeing his behavior we thought it may be superstitions like some black magic or some bad omen but she told us to take him to psychiatrist then we took him to bhimavaram hospital. Doctor asked him about problem. He used to see in different way he was not eating food he used to cry usually as we are worker we don't take care about all these thing when we have headache we take paracetmol and amruthanjan or we drink tea that's it but we never heard about these mental health problems after seeing his position we were quite depressed. Now he is taking treatment and now he is fine

I: You madam

P5: I have heard the same case

I: I mean in any other place

P5: I have seen this in other place too my niece also suffering from such a kind of mental health problems. She used to run away from her husband, she was taken to hospital her hair was removed operation was conducted we don't know that such kind of treatment exists but she taken her to hospital at that time now she is completely fine.

I: If there is a person who is suffering from these kind of mental health problems then how will you identify him/her?

P7: As we know there behavior if we see any change we will inform you if you give your number

I: If we are not available you got doctor in PHC you can also approach him

P2: symptoms like he/she wants to be alone and they will be always thinking about something

P8: So by seeing these symptoms our people think that it is the effect of ghost or devil

I: If 2-3 person in your village are suffering from this mental health problem then how the people of your village treat them? we can see in the films like they make fun of them or they will insult them so how your village people treat them?

P6: we don't know about it after seeing these cases we came to know that these kind of problems can also be there. So if we see anyone like that we will try to help them and also give moral support.

I: If a person is suffering from mental health problem some people prefer to take them to baba's or faith healers so have you seen any kind of these behaviors in your village?

P3: No

P2: If a person suffering from mental health problem as a human we must never make fun of him/her if possible we must help them

I ; You madam…

P: we must give advice or reference to doctor as we came to know now will we definitely take them to hospital for treatment. If they are not ready to come to hospital we will inform ASHA's or doctor about him/her.

I: You madam..Do you think it is a problem?

P5: Yes it is humans must understand each other….

I: Your opinions…

P4: We will refer them to doctor if we observe any one.

I: Which kind of facilities are there in your village for the identification and treatment for mental health problem in your village?

P3: There are no facilities for that but if we approach doctor he may refer to the right place for that problem.

P4: we have only one PHC there are no facilities in here for mental health problems they treat fever and other physical problem but they usually refer to some other hospital.

I: So as we talk about mental health problems what should be done in your village to increase awareness about this mental health problems?

P5: Meeting must be held like this. I felt bad that you came for us to care for our health then next time when you come here organize a big meeting where everybody will come to hear you.

I: OK Meeting….Any more ways apart from meeting? We want to know a easy way to convey our message listening about mental problem people think in different ways about it they may feel awkward to attend meetings like this.

P2: we have to conduct a survey to each and every house

I: who must convey this message so that everyone of your village follow it?

P3: You must say we will listen you

I: Apart from us because we came from outside and if we say something in this village it may not be effective persons who belong to your village must speak like president or head etc., is there anyone like that?

P5: People don't listen to us. Persons who come from outside like you must speak because they may listen to you. People think that this madam came from Hyderabad to educate us about mental health problem so thinking like this most of the people listen to you.

I: You madam..You are not speaking anything

[cross talk]

P6: If you conduct a meeting on Sundays we may come to listen you

I: Will they not listen to us too apart from meeting?

P1: You have just asking questions based on a structured questionnaire but it is not the right way…. anger, anxiety, tension, fear, quarrelsome nature are also one of the mental disorder..

I: Yes I agree with you but if we tell you about mental health problem and details we will not be able to know that how much you are aware of this problems. so after having discussions with a group of people one by one we may get some idea that where we should start from.

P1: Anger, doubtfulness, anxiety is also disorder and you must say ways to rectify them and also must enquire if we have seen any cases like that. I mean that you must name some of the disorders to make some awareness regarding these problems.

I: You madam do you want to add anything?? she means which kind of media must be used to increase awareness?

P4: discussion is one way where each everyone will participate and the news will be spread once they meet 10 people they will share the same experience or information that they gained through these discussions. So group discussion is good means of spreading or creating awareness.

P5:Though some people may not be aware of these conditions but they might get information as they trust us.

I: Where should we conduct these kind of programs in your village? Where is it convenient for people to attend

P3: I feel such meetings must be conducted in each locality in the village.

P5: In panchayat office all may not come so each area wise is good ,can conduct in church too all may come.

I: Thank you so much

P: Thank you

I: As they already said the main reason of coming here so we have opted some ways to create awareness so for that we will come again and will conduct a program for your welfare.

FGD 3

Date: 22 Jan 2015

Place: Village[Name…..]

Group Details: Males

Moderator: Mr. Siddhardh Devarapalli

Note taker: Sudha Kallakuri

Moderator: A very good morning to everyone. We are very thankful that all of you have come here to discuss about the topic of mental health knowledge and awareness in your village. Before we start I would like to introduce myself. I'm Siddhardh and they are my colleagues Sudha and Balaji. We have come from a not for profit organization called George Institute for Global health, Hyderabad. We are visiting not only your village but also other villages in and around the west Godavari region. As I mentioned before we are here to know what do you feel about mental disorders. I request you to kindly give your valuable inputs which will be very useful for us to design an awareness programme. We also would request you to speak one by one and keep your mobile phones in silent mode. If you have any queries in between the discussion please wait until all the discussions are over so that I can give you ample time to answer your question. Please do not laugh or ridicule anyone's response as every individual is different and everyone's perception is different and it is important to respect them. Also please speak one after the other for responding to a specific question so that all the answers can be well heard.

I: To begin with what do you think is a mental disorder?

P3: People will not be able to do their regular activities well. They just roam on roads and talk to themselves.

P1: They usually do not talk to anybody. They are in their own world.

I: Ok, So did you ever see anybody suffering from such disorders in your family or in your neighborhood?

P: No there is no person like that we never saw

I: You…Actually the mental disorder

P2 and P1 : Yes we know of a person who has a deformed finger due to some injury while he was working with a hammer, he spent a lot of money to get treatment for that finger so I feel this is some kind of mental disorder where he keeps thinking about that finger all the time.(together)

P3: Sir, Can you please tell me if there is any kind of dizziness involved when a person takes medication for a mental disorders?

I: No… All mental disorders do not require medications.

P3: For instance most of the patients go to a hospital in Bhimavaram and when take medications prescribed by the doctor Mr. Ramana Rao they feel very sleepy and drowsy all the time.

I: One must note that this varies from case to case and all of them will not be prescribed the same medicine.

P1: In such cases people their mind gets diverted and hence I feel if a person is given these kind of medications because of which he feels drowsy or sleepy he will be at ease otherwise he will start behaving abnormally.

P4: Sir you are discussing about mental state of a person, for instance a person might be absolutely be normal at birth but after he attains a certain age he goes in to depression what do you think is the reason for that? Till now in our village we haven't seen such kind of cases but now we are seeing a person within the panchayat circle. He might be around 35 years and before 2 years he was fine but now he talks with himself he looks differently. He blabbers something because of which people do not understand if he is abusing someone, so what might be the reason of this kind of behavior?

P2: He does not have any addictions like alcohol.

I: There might be different reason. Generally perceptions or the way people think varies from person to person. For example if you are in a problem so to solve it your family may support you knowingly or unknowingly. Apart from family you might have seen some people share their problems with someone who is very close to them.

P5, 6 left in between the discussion

I: Especially when you are in problem first you have to share it then when you discuss the problem with other person you may get some solution. Some people might not like it and they don't follow this because they don't like to share that problem they keep it in themselves so by doing like this problem increases.

P4: Yes you are right this might lead to depression.

I: That's the reason we included trained doctors in this project because they mainly advise to share problems with other to prevent so mental stress, because the main motive is to get a good advice for a problem which might give ease to a person and not any kind of advice. And hence when a person discusses these issues with a doctor he will be in a position to judge how serious the problem is and depending on case to case basis treatment will also differ like it could be just counselling, or advise of medication or combination of both. Please remember sometimes a small advice can change your mind or in some cases medication may be necessary it may start with a small course of medicine not as a bunch of medicines so depends on person's problem medication can also be prescribed.

This is what we intend to do in our research study that we plan to do. In this process we will also be involving ASHAs who's job is just to identify the problem and refer the patient to doctor who

will do the further diagnosis and treatment. We will also go intermittently to the doctors to check if this process is working.

I: Ok so, when was the first time you heard about these kinds of mental disorders?

P1: Yes we have heard of such cases in our villages.

P2: Yes there are such issues we are recollecting as you explained just now. But not more.

P3: We have seen 2-3 cases in this village.

I: NO, by mental disorder means the mental stress, tension which we usually face in our daily life…

Everyone has these problems like tension and stress in their day to day life (All together, laughing)

I:Yes That's what I said though everyone has these problems we need to identify to what extent a person is facing stress or is getting tensed. Related to this I quoted an example aIso where we need to differentiate a person who is getting tensed for a particular problem and who is getting tensed for each and everything he does. So we need to identify such people. You had asked me earlier that why people get depressed so it may be due to failure of identification of such issues in such people. Because of this failure the problem is getting serious and so our research study mainly aims at creating awareness to at least identify this as a problem. We are trying to find out if people actually recognize this as a health problem.

I: Ok alcoholism, ok that is one problem

[Cross talk all of them talking together]

P7: Ok sir can you tell me you have come all the way from so far to a small village? What is the reason of visiting our village?

I: We want to link up these kind of problems to government with PHCs. For instance in PHCs currently mental disorders are not WHO has recognized that there are lack of trained specialists in the LMIC to treat mental disorders. Hence we want to identify and train the health professionals at the PHC level in a village set up to treat mental disorders in the given set up.

P3: Yes, as yours is a voluntary organization so take it to the notice of government you have to bring change in people and bring change in the society as well. We don't need to give employment to people, we don't need to give subsidies.

P6: So sir, can you please tell me don't you need to take help from the local health organizations or voluntary organizations as they will be more aware of the situation?

I: Why? We have come through PHCs isn't it? We came here to know how much people are aware of these kind of mental health problems.

P6: Oh it is only up to that much!!

I: No its not only that much, based on the information that we collect about the kind of awareness or knowledge that we have about these problems, we will get help to plan further programs on creating awareness in that particular locality. For instance when I talked about mental disorders all of have emphasized that alcoholism is one of the main mental disorder. In a similar way I would like to add that anxiety and depression also are considered as mental disorders. And these problems if not recognized at early stages can become very serious issues.

P3: Sir one thing I can say, mental problems like anxiety and depression are very rare in our village. The whole population of our village is around 500 and I feel the most burning issue here is that most of the people are addicted to drinking!! So please do something and try to reduce it. And from government's end what can be done please think only about it and keep all the other things aside.

I: As you said that alcohol is the main problem in this village; but that is your thought

P3 and P7: No, not only in this village but also in other villages too…

I: Ok please let me talk. Whatever we say that is after we do a survey and we collect some data and based on that we come to each and every place. Drinking is also problem but along with this problem also there are other problems.

P: As you went many places what kind of problem they have?

I: Intentions to committee suicide was another problem that we saw not only in middle aged or in old age people it was seen in youngsters also. We got to hear about this when we went yesterday to Pedhanindru Kolanu. What you are saying that anxiety and depression are not at all a problem, they said that as major problems.

P3: So, don't you think alcoholism was the main reason for committing suicide in all those cases?

I: No, Alcoholism need not be the root cause. They are college going people.

P3: No why I'm saying is….my son….He also committed suicide but…

I: Yes, but. .

P3: He drank much alcohol that day when he committed suicide

I: Yes you are saying that in your village alcohol is the main problem but cases may differ depending on villages for example If a married women commits suicide she may have problems

with her husband or due to her in-laws and the students who are studying might be feeling so stressed out due to studies and pressure from parents and the teachers which is also giving them tensions, because of these reason they are trying to escape from this stress. Some are choosing suicide as one of the way to escape from stress. Yesterday itself we got to hear about a case study in Pedhanindrukolanu, where a boy who was was studying medicine in Vizag, tried to go into beach to commit suicide and his friends saved him. So tell me where is drinking the cause of this act in that case not only him there was also another example shared by another lady where she was also telling the same problem especially these kind of problems are getting increased in students and there are feeling so anxious which is a problem !!

P3 and P4: Ya these cases might happen but I think Percentage is low… (Together)

I: No, percentage may be low but life never comes back it is precious it may be a single person. In one village even if here is one such case we have thousands of villages if a person dies from each village how much the percent will be??

I: So what I intend to say is the alcoholism is also an important problem but apart from that there are many mental disorders, the first problem is to identify these kind of mental disorders.

I: First of all we have to identify this anxiety, depression, and stress and alcohol consumption as a mental disorder and how do we tackle them. We are not ignoring alcoholism as that is very predominant in a village set up and avoid concentrating only on alcoholism as it may lead to an increase in other mental problems. So considering these factors can you please tell me how can we convey message to people about mental health or to reach them to bring awareness about these problems? Ok you tell me in the context of alcohol itself?

P3: Sir shall I tell you something, We have this guy who consumes alcohol every day, he doesn’t listen to us if we say to stop drinking so if a new person says him he might listen to him.

I: Based on this point can anyone explain it in detail? Which kind of person must convey message?

P3 and 6: Medical doctors from government, some voluntary organizations like yours should come to gather some kind of evidence and submit to the government. Mobilization needs to be done to reach out to the people.

I: Ok, so how can we do the mobilization?

P4: We must create some kind of fear in people who drink alcohol.

P7: Ration facility should be removed for people who drink alcohol.

P1: Government must stop giving license to these kind of bell shops etc., it must put some kind of pressure on every person.

P3: For. E.g this recent swach bharat initiative, government has made it compulsory to build toilet mandatorily in their house in case they are building a new house.

P1: Recently there was a General body meeting where it was informed that the houses which are built must compulsorily have toilets if not they will not sanction permission to build house a special officer came from Eluru that day they said that every house must have toilets. In a similar way alcohol problem will also be tackled.

P3: In a similar way none of the Governments formed so far have taken steps to eradicate alcoholism and problems associated with alcohol consumption so far.

P1: Senti Raju garu, sir told before that each one of our opinions will be noted and our opinions will be studied in detail and they will come back to us so please tell them your opinions…..

I: Yes, Your individual opinions are more important to us

P: If because of this program if we succeed to bring change in 10 people it is enough for us. It might be one of the good things that we ever done if we bring this kind of change in head of family; it is like giving that family a 10 thousand rupees as salary per month.

I: Are there any more opinions?

P1: It’s difficult

I: tell us. .

P1: Many of the adults are addicted to alcohol now – a-days and they get affected with diseases like HIV. This must be prevented and also I heard that there is a new kind of fever out there(swine flu) where I heard some nurse are giving medicine for it but we didn’t get it yet so can you help us in that matter?

P3: Swine flu cannot spread here it is getting treated maximum

I: Ok, that is a health problem but can you give any advice regarding mental health problems here?

P1: No, about mental problem I think as due to too much consumption of alcohol they may be facing financial crisis and they think about it and because of which sometimes they beat their own wife or they may get in to activities like thefts etc to pressure.

I: As you said just now that some people may beat their wives so he might beat her due to alcohol but what do you think might be his wife’s mental status…don’t you think she might be depressed about it?

P: Ya, also I feel the person who has beaten his own wife may also be suffering from mental health problems.

I: About her…She might be so very disturbed each time her husband beats her. Isn't it!

P2: But the treatment must be given to husband to solve this problem

I: Yes that's right he needs treatment

P3: I have seen a person recently; he was fully drunk he beat his wife and also send her out of the house at night 10'o clock and during that time she fell on my bike and got her hand fractured.

P1: She undertook a treatment for a period of 30 days.

I: So imagine how much effect it would have had in her mind?

P2: But she adjusted with him still he didn't change.

I: Consumption of alcohol is serious problem but that effect also fall on family and they may also suffer from mental health problems so we have to identify it as a problem then only her mental health can be fine.

P3: No I am talking about him; Sir both of our views differ here only.

I: Yes drinking alcohol is also harmful but you are only considering it as a main problem but I am considering both problems a main ones

P4: Are!! sir is is saying that she must be treated to bear with him

I:(Laughing)……..No not to bear him both of them need treatment

P3: She doesn't need any treatment she is fine but he needs treatment

I: As he is addicted to alcohol we must consider it as problem and we must compulsorily give him treatment

P3: No do you have treatment for these kind of alcoholic people or not? I just want to know about it

I: This discussion is not only about alcoholic people

P3: Mental health problem is nothing but addiction

P7: No no he is talking about different kinds of stresses.

P3: But as a voluntary organization can you do something for alcoholism?

I: Yes, We will also give solution for these kinds of addiction problems that's why we are talking about it. If I can give another example if we take pregnant ladies some of them also suffer with

some kind of mental health problems have you observed it? Usually from the first day of her pregnancy they feel tensed and excited

P3: Yes but doctors give suggestion to them so the problem will be cleared to the maximum extent in most of the cases.

I: We think that their problem will be solved but it won’t.

P: We haven’t seen any pregnant women suffering from mental health problems in our village.

I: What do you mean by mental health problem?

P: Pregnant ladies may feel tensed because they are worried about their delivery whether it will be an operation or normal delivery they are more concerned about it but doctors give suggestion to them about all these kind of things.

I: That’s what I mean as they go to lady doctor they are given suggestions that’s what I am also saying as you have understood about the tension and anxiety in pregnant ladies in the same way student and all the other adults may also have different kind of mental health problems so we must know about its importance to identify It and bring awareness about these what should be done?

P3: I told about alcoholic problem because in our village every problem has some or other link with alcohol

I: I Agree but….

P7: If drinking is banned government will collapse

P1: No How can be the government be effected by it?

P1: Bikes, Lorries, bus will stop running on roads

[Cross talk]

I: Sorry but we are diverting our topic which is mental health problem so please talk about it

P3: No I don’t like it because you are only talking about mental health problems but there are many kind of problem out there, for example B. P and sugar

I: No sir we are conducting survey from 2007 on many health problems; not only on this as a part of different initiatives taken by the George Institute.

[Cross talk]

I: No, No let me tell you in detail as you do not have an idea about our institute

P3: You are only talking about mental health problems

P1: He is telling that they conduct survey on everything listen to him

I: We didn’t tell you what George institute has been doing, so it is conducting survey on B. P, sugar and also on mental health problem. As many people are not taking these kind of problems seriously we are here to find out some solution to these problems and as many people are talking about sugar and B. P problems then there is not point to again talk about it as the research on such disorders is already going on

P3: No but you must at least suggest medicine and health diet to those kind of diabetic and b. P patients then there may be chances to reduce these kind of problems?

I: Not only has our organization done research on sugar B. P but there was a study done on how salt consumption can worsen the health was also going on so along with them we want to know how the people are taking these kind of mental health problems and also want to know how much you are aware of these kind of problem as you said that many people must share their problem but some don’t like to share so how can we treat that particular person who doesn’t open up about his/her mental problems? So we want to understand the scenario of mental disorders in a village set and hence we have come here.

P3: As I said earlier, wife gets adjusts with her alcoholic husband even if he beats her everyday. As you said she might keep thinking that why he is beating she and she may commit suicide; but such cases might be very low in our village.

I: No, I’m referring to the example that you cited some time ago about a person earlier….who keeps talking to himself and behaves vierdly…..

P3: In middle class family many men drink but they don’t beat their wives such kind of cases cannot be seen in middle class families but in lower class people we can see that kind of behavior

I: No you said about a person earlier that he is talking to himself like that so for those kind of people do you have any treatment available in this village?

P3: Most of them go to psychiatrist

I: Is there anyone nearby your village

P3: No, most of them go to Bhimavaram

I: Ok, Bhimavaram

P: We don’t have specialists here

I: That’s what the problem is here now specialists are less in number so to solve this problem in our PHC we want to give training to doctors so that they handle these kind of mental health

problems. So already your doctor in this PHC Dr. Harish has taken training regarding these problems. He was specially trained on how he should handle these case and how should he identify such individuals with mental health problem.

P3: Ya, what will he do, He will just look at patient and refer to psychiatrist Rama Rao (Laughing)

I: He cannot refer to Rama Rao as he is a private doctor

P4: Yes he is a private doctor as we don't have govt. doctor near from us

I: That's why we have given training to them and also told them where they have to refer the patients

P: Is there any psychiatrist in our district hospital?

I: Yes we have one in Eluru, which is the district hospital

P1 & P3: If we go to Eluru for treatment will it be effective and feasible for all of them to go to that place?

I: Ya that's what; If there is a psychiatrist in district hospital then it is not enough so we have given training to doctor in PHC only for people's convenience who can't go so far for treatment and we are also trying to make medicine available to you especially in PHC. we don't want you to spend more money in private hospitals our aim is the people who are suffering with mental health problems in villages we want them to get help from government PHC's.

P3: My argument is nothing but making our village people to stop drinking is the only solution for every problem in this village does it with the help of government or through George institute

I: You tell us what should we do to solve these problems?

P3: You inform your organization and follow their instructions

I: no they will not give any suggestions; we came here to know your opinions

I: What do you think you must do for this kind of problem?

P: Awareness must be brought in people; Take for instance about Dwakra groups; we earlier din't know about Dwaraka women but now we know that they have created some consciousness among women as they go here and there and hence that can be one of the strategies. They must also have some awareness about it, so first the wife of an alcoholic must get some awareness.

I: Dwakra women and any more. .

P1: Private or government hospital treatment must be given to such kind of patients.

P3: We must bring pressure from government

P7: Say for e.g. if my shop is removed by government now; if we want to have alcohol we have to go 4 kms far as they can't go every time they have an urge to drink many people quit that habit if it is near then many people are even going to an extent of taking debts to drink alcohol.

I: Any more, any other ways

P7: There is a place where some of the alcoholics from our village went; people say that they give bracelets and the person who wears it must not drink if he drinks they say that he will die so many person quit alcohol consumption as they got scared of these kind of rumors. Some people who have stopped this for a period of 1 year completely quit drinking because of this fear.

I: Who got this idea of bracelets?

P4: There will be some circle of people in and around the village where people keep talking to each other that this person is getting addicted; so this has got initiated from such people. Also you must be watching some specific adds on TV where they say if you have any problems related to alcoholism please come to us etc etc…

P4: So ultimately this is more like counseling which is 90% and the bracelet effect might be 10% only

I: Have you observed this that as you said that counseling is 90% and bracelet effect is of 10% so this is what we also focus on, it depends on the person who says and also the person who listen to him. for example if a person have intention to quit the alcohol then only he gives important to the person who is talking about quitting of drinking habit so here also same thing applies. if a person is in any kind of mental health problem counseling must be given which has major impact so medicines are not important in the case of mental health problem those are the next step as medicine may also be necessary depending of the case complexity and sometimes counseling and medication both may be necessary in some cases problem may be solved due to counseling but the thing is we have to identify it as a problem.

P1: Many people may come to counseling if we give them a bottle of whisky (laughing)

[Cross talk]

P1: It also depends on the way of counselor

I: IS everybody going to that bracelet place if they are given a bottle of whisky?

P3: No nothing like that.

I: Is it happening in your village or in any place

P1: Somewhere near to the village we don't know exact place

P7: In Rajahmundry

P3: You went to Rajahmundry does it work?

I: Its cost is 5000?

P2: Yes

I: So if we want to conduct an awareness program how must be that program organized for you?

P3: Doctors team must come here along with politicians as now they are becoming center of attraction. People may not come if we and you call them if they see any politician they may come so this kind of 4-5 teams must be in action to run these kind of programs. Attention must be drawn to make them listen to us.

I: Any more like you said doctors, politicians…

P3: It is enough

I: If for any other program then?

P1: Our message must go to door to door to women and men in a family and they must be given some moral support if they identify any kind of mental tensions it can be cured etc. , so these kind of things must be done

P3: Awareness can't be brought up when such a kind of volunteer services come up for once in a month they just come and go

I: So what must be done?

P4: It is also difficult to bring awareness through a meeting as he told that we must go to door to door and must also play something on screen

I: On screen…u mean like a short film?

P3: If we can let the people know how the consequences of these problem he has to face due to these kind of habits.

I: Any more…So as you might have seen some kind of documentary when we got movie….something similar to that?? is it enough?

P3: It may need more explanation

I: Ok

P4: Counseling is also necessary

P3: If a husband is beating his wife there must be a committee when she complains; action must be so whenever she threatens her husband he must be scared.

P1: Only government can do it

P3: I can give you an example; when a husband was beating his wife on the road at 12 a. m I was there; I threatened her husband and in-laws as they are not getting inside he got scared when he heard police station name….so similar things should be done.

I: Do you have any more opinions?

P1: government must do something to prevent these kinds of addictions

How can we do?

I: We will make everything implemented through the government

[Cross talk]

I: What kind of program must be conducted to quit drinking?

P1: He may listen to police

I: You…So thank you so much for giving your opinions we are going to conduct surveys in villages under Pedhanandripuram PHC so by taking all these opinions we will try to implement some of your ideas to create awareness among people about mental disorders; though today we have discussed more about alcohol.

FGD 4

Date: 22 Jan 2015

Place:Village [Name….]

Group Details: ASHA workers

Moderator: Mr. Siddhardh Devarapalli

Note taker: Sudha Kallakuri

Moderator: A very good morning to everyone. We are very thankful that all of you have come here to discuss about the topic of mental health knowledge and awareness in your village. Before we start I would like to introduce myself. I'm Siddhardh and she is my colleagues Sudha and Balaji. We have come from a not for profit organization called George Institute for Global health, Hyderabad. As I mentioned before we are here to know what do you feel about mental disorders. I request you to kindly give your valuable inputs which will be very useful for us to design an awareness programme. We also would request you to speak one by one and keep your

mobile phones in silent mode. If you have any queries in between the discussion please wait until all the discussions are over so that I can give you ample time to answer your question. Also please speak one after the other for responding to a specific question so that all the answers can be well heard.

I: Mental health and its problems, what is meant by mental health its symptoms and all other details can be know after sometime, what do you think the mental health is what kind of factors make affect on mental health? What do you think the main factors are for causing mental health problems?

P: If one who is suffering from tensions or due to the death of the dearest person or the person is suffering from financial crisis these all reasons may make person mental condition weak

I: Any more reasons?

P: There are many

I: Continue what are the factors which you think that can affect mental health

P: Everyone face problems in their daily life usually more people suffer with health problem

I: You mean due to health problem or due to sickness may also affect the mental health?

P: No only for us if any family member is suffering from sickness may be sister, mother or husband anyone it may also disturb us mentally

I: So this can also show affect on mental health…Ok You madam

P: Due to problems in family or pressure can also affect mental health

I: What do you mean by family problems can you explain in more detail?

P: Quarrels between wife and husband or between mother-in-law and daughter-in-law and also due to health problems or if a person is humiliated then it makes person feel sad

I: Anymore.. You

P: In my home there is lot of problems..

I: Not family problems…

P: My sister's son want to be a doctor so he used to study more and more books he will be just always with his book his mental condition was not good we thought he may die but we have taken him to bhimavaram hospital treatment was given and now using medicines too apart from it there are also some problems

I: What kind of problems?

P: Due to children my elder son fell sick and taken to hospital for treatment and my younger son had a tumor in this brain so I was disturbed a lot at that time I didn't used to eat food for 2-3 days by thinking about all these things and my parents also not feeling well my father was suffering from diabetes due to it his leg was amputated at that time my elder son also fell ill I have to take care of both of them at the same time my father's operation was at night he wanted to see me once and here my son needs my care I told all my problems to bhayga laxmi she is my friend we can't share everything with parents right? We can share some with our friends my father was died and my elder son needs treatment I believed in god and faced situation firmly elder son was fine but my younger son died due to t.b in brain my mother also suffering from health problem and my sister have 4 chidren 2 boys and 2 girls her husband is not good but my mother says to her not to leave him and just manage there because is may damage the prestige of the family if she leaves him so this effected her mentally and she used to beat her children we taken her for treatment manyam beyond rampachodavaram we requested our mother not to give stress to her then we taken my sister's husband to police station after that his behavior is good and she continued to live with him, we have faced my problem and mental tensions too but I never used medicines I trusted in god after my father's death family was in much loss but we handle our mother well and also give her advices too if she quarrels with her daughter-in-law we tell her not to interfere in her matter and whenever we go there we also don't interfere in their matters

I: You madam

P: we are suffering from financial crisis and my daughter go married 6 years ago they don't have children and my mother is also suffering from B.P and sugar if we say any kind of bad news to her she feels so tensed and worried so we usually don't share anything with her

I: How a mental health problem is considered in your village?

P: If a person is suffering from mental health problem here they take them to a person who gives tabees and usually considered as a ghost of a devil which has entered in to that person who is mentally not well, but that is not the right way the person must be approached by this dear one like friends as we share everything with our friends a friend must approach that person and must know the person of his/her behavior like if a girl is not agreeing for marriage her dear one must know the reason of her restrain and also about her opinions

I: Not opinions..How mental sickness is treated in your village as you said that some consider it as intrusion if devil/ghost so any other considerations?

P: They usually take to superstition physician they give thread and other thing and if after using that if we don't see any change then they think of mental health problem and they go to hospital for treatment

I: When a person is suffering from mental health problem what do people think of it do they think that it can be cured or not?

P: Yes we can give advice or reference of the doctor where it can be cured

I: I mean here in your village do everybody think that it can be cured ,Do they believe

P: Yes, when we explain clearly about the problem then they listen to us

I: You mean that people are ready to listen anything about it?

P: Yes, in those days they might not listen but now they will listen to us

P: There is a person who was fine he used to do prayers and all but suddenly we don’t know what happened to him he used to pluck his own garden and he used to climb on wall and cut down tubes and pipe and after sometime he used to shout that who did all these things he also use to say that he was suffering from HIV and he needs treatment then he taken treatment now he is fine

P: Now he says prayers in church usually he is completely fine

I: As you said that some people believe in superstition things and goes to treatment there so there are many people in our society who still believe in these thing so what do you thing we must do to stop things

P: We must tell them smoothly if we say that person is mad they may feel bad we must handle smoothly and must take them to hospital

I: the person who is suffering from mental problem must be approached smoothly and must taken to hospital

P: When we observe any change in their behavior we must take them to hospital for treatment we must give moral support to that family

P: Angan vadi teacher son climbs up and tear down the book he used to call us sister but after sometime he failed to identify us his parent was worried about him then we advised them to go to bhimavaram psychological hospital for better treatment same was happened with my sister’s daughter too after her marriage her behavior was changed she used to beat her mother badly and also used to drop kerosene on her mother’s feet an after a moment she used to ask who did it so we taken her to Rajahmundry hospital she was so violent and also used to beat nurses but they have given good treatment now she is completely fine in olden days they used to believe in ghost and devils but now if there is any mental health problem they are going to hospitals there are very low cases in here mental tensions are due to children, financial problem etc

I: Not only these advanced cases but also initial cases like the people who feel anxiety, restless which can also be considered as mental health problems like drinking or suicide ideas and attempts can also be included so for these kind of problem do you believe there is any kind of medical support available out there?

P: Yes we believe in it

I: If you believe it then what do you think that in how many ways the treatment can be done, In how many ways can we treatment those kind of mental health problems?

P: we must give our moral support to that family and we must talk in a friendly manner to that person who is suffering from mental health problems and must refer him/her to the doctor for better treatment

I: you madam

P: Same sir

I: Means is there any other means to give medical support meeting the person personally and giving counseling is the advice one given by you, so is there any other support which we can give to those people

P: The people who are mentally..

I: NO, I mean not only those kind of people normal people with these kinds of anxiety and restlessness so how can we give medical support to them one is counseling and any more

P: If it is in high range then we must refer them to doctor

I: If it is advanced then they must be taken to doctor ok…any more if in the case of normal mental health problem is there any other support or advice which must be given in the case of treatment?

P: health advices can be given..

I: In which way?

P: the person who have drinking habit must be advised to avoid it

P: [cross talk]

I: How can you convince a person to avoid drinking

P: By telling him how much his family suffers due to his drinking habit

I: Your opinions madam any more ways for health care

P: We must give moral support and also advice to them

I: your opinions madam..

P: when we see any person who is suffering from these kind of mental tensions we must advice him/her not to take more stress and we must also explain what may happen in future by taking more stress and tensions

I: You told about injection can you say more about it?

P: A person's drinking habit can also be avoided by using some kind of medicines if a husband have his habit we must advice his wife what to do and also the husband to agree for treatment

I: You were saying something…

P: Yes, usually cigarette [lot of disturbance]

I: Speak louder

P: The people who smoke must also be advised and if they don't listen they must be sent to doctor

I: You mean if tell them the consequences of smoking like cancer then it may lead them to leave smoking..

[lot of disturbance]

I: Was there any awareness about these kind of mental health problems?

P: Awareness means we have seen 2 cases like this so we do have some awareness about it

I: Ok, and what about other people?

P: After seeing her son then we came to know that these kind of mental problems are also there

I: You are from ganapora

P: A girl was not feeling well then doctor given advice to make marriage to her she is fine after marriage now she has2 children one of her cousin has nerve weakness he was also married but he was still suffered from nerve weakness he also have 2 children his mother has committed suicide and after having 2nd child he committed suicide

I: Is there any easy way to get medicine in our village

P: Yes through doctor we can get it

I: Can we get easy treatment and medicine for these kind of mental health problems here in your village?

P: NO, there is only one PHC we can't get treatment or medicine for mental health problems here

I: Now at present

P: Means most of us go to private hospital

I: Going to private hospital makes money loss then it can't be considered as easy way

P: Means the people who can afford will go there if not they don't go for treatment

I: Yes, the people who can't afford remain at home so there is not easy way to get treatment for them

P: There is no easy treatment

P: As you came now there may be..

I: Up till now

P: Here we give advice to pregnant ladies and new born babies we take care of them we can give advice that's all

P: we usually deal with operations, health care of mother and child

I: Do you think that there must be some awareness must be brought about mental health problem?

P: Yes, compulsorily it is necessary

I: What must be done to bring awareness in people?

P: Now as you have come in to villages you have observed many in the village if you divide the people among us then we will take care

I: What must be done to bring awareness any more ideas?\

P: Meeting must be arranged

P: Meeting must be done area wise like if we call harijan pet people they may not come here so we have to keep a meeting in their area so that they can attend without fail they won't listen us they will listen you if you say anything

P: Everybody will be available at Sundays they can attend [cross talk]

We usually conduct in school and we also announce to people

I: You mean meeting must be done ok if we arranged meeting with along with your sarpanch then who must talk there like who's words work more on your people

P: You must talk

P: Doctor’s advice can work good , you and doctor must talk then they will listen to you

[cross talk]

P: before one day announcement must be made we have school there we conduct most of our programs there you can also keep meeting in church

I: If we say this in panchayat office will they help us

P: Yes , they will

P: It will reach everyone

P: President can help you he will arrange

P: They will listen you and especially on Sundays meeting must be conducted

I: One more thing…You say that these kind of awareness camp must be taken to home?

P: I mean some people may can’t say openly about their problem so some may share some may not

I: Meeting on mental health problem must be done by the doctor then it can reach the people is one opinion and the other is to go to each home so that the people who can’t share problem in public may open up personally, Any more opinions

P: doctors must say it may be effective I other people say they may don’t listen

I: there is difference

P: there no awareness

I: Thanks you so much for sharing your opinions with us

Interview with doctor

Date : 23 January 2015

Name: Dr [Name….]

Place : PHC

I: I will start asking you question one after other now

P: Ok

I: Can you please tell us in your clinic or in society the patients suffering from mental health problems belongs to which community?

P: Mainly old age people who are more than 55 years most of them are care lacking persons like their son may not take care of them like that kind of cases are more

I: You mean to say that due to financial crisis

P: Those kind of cases are also less not due to financial support due to lack of emotional support

Like when son get married he might take care of his parents they may feel lonely so these kind of cases are more main thing is they feel humiliated they never share it but during treatment they open up if they are suffering from headache I usually ask they do they think more then they open up about there feeling so 50% of them don't talk about financial crisis and 30-40 years age females say about there family problem about her husband who is drinker or smoker some of them say that they were constructing new house and they are lack of money so less cases come up with depression due to financial crisis

I: So as you told that normally the people above the age of 55 years are mostly suffering from mental health problems

P: Yes

I: In the same way have you seen any person who is severely suffering from mental disorder

P: No I haven't seen any person like that here mental depression situation is called niyonic but I have observed here is if any person kept on waiting here or if other person takes his place they shout out that is only that thing I observed here they come here mainly to take medicines as we give medicine here right ? so as a to take medicines or if other staff bring some more patients they may go first so in that situation they shout out

I: So you mean you have observed more CMD's here and you haven't come across any SMD's

P: Simply there are no one

I: Okay.. You means to say that the people over 55 years are suffering from anxiety and depression

P: [unclear] lack of care, sometime financial crisis and severe stress means they come here they take medicine and make checkup of their BP they are not willing to talk much and if we ask particularly then they open up and they try to share their problems they particularly come here for treatment and medicine so usually they won't reveal their problem so when we ask about it then they open up

I: You take lenience and try to observe if they are suffering from any mental disorders

P: Yes

I: Then they share with you

P: Yes they feel free if we ask anything like that

I: They feel some emotional touch as if we ask about their problems and if we share it

P: Yes

I: Is it comfortable to you to give treatment to the patients of mental disorder in your practice?

P: There are no such cases here more over some people come with the complaint that they are not getting sleep even I don't prescribe digipomo if I observe it CMD problem I ask them to go to some higher hospital but till now I haven't seen anyone clean case of CMD problem

I: Ok, [cross talk]

P: They are using ultraxen, restin now these medicine became compulsory for sleep or any kind of other mental disorders

I: As there are no resources for mental disorder treatment so you refer them to other hospital

P: Yes I usually refer psychiatric doctor but they feel awkward to go there so I refer M.D

[cross talk]

P: I ill observe the persons complaint and try to identify if the case is CMD or a normal stress if it is CMD I try to get him maximum information

[cross talk]

P: Mainly CMD people look for relation so if we maintain good relation with them they automatically come to us to share their problems

I ; What is the main problem you faced while talking to them?

P: The cases which I have seen till date was due to lack of emotional care at too in old age people and in adults they face positive cases they afraid that if any disease my get their due to hereditary so they think more and go into depression like husband has HIV positive so there are chance the child may also get affected by HIV so they think too much like this

I: So they remain in this feeling

P: Old age people problem is lack of care and like widower or females are suffering from mental disorder due to lack of concern from husband that's it most of these common cases come across

So they also don't come regularly they come after long time

I: So the people who come here with the problem is also less and if there are such cases you usually refer to other hospital I asked to know that if you have faced any kind of problem while treating mental disorder patients

P: Most of the people won't open up freely so we have to be friendly to know their problems

I: What do you think that how these kind of mental disorder problems are considered as?

P ; Our Asha group mainly concern about old age people like one may be against of other's will they won't permit the other to do what he want so behaviors changes according to age married people like they won't do what her husband want to do and some may won't allow their children to go out to play or for movie so behaviors may change due to age

I: SO generally there are rumors that by visiting temple or baba's or superstition persons may cure mental disorder what do you say about it?

P: Some persons believe in religion and they think it is only the way to come out of problem so usually we meet everyone they talk with each other some may say that they went to a temple and came out of problem and this will spread among the group and everyone start doing the same thing they must know that by sharing problem they can feel better

I: So what do you think about superstitions treatment?

P: I don't know about them but i have heard that they good treatment rather than us for snake bites they say and the funny thing is a woman was bitten by snake she don't know I too don't know but that superstitions person observed it and told it as snake bit I don't know how he told it so then they asked me for treatment and treatment with those kind of magic is ridiculous I think our trust is there capital as so many trust them they are taking advantage

I: Have you heard any rumors about mental disorder?
P: No, but

I: I mean they may be humiliated or they might be treated in different way?

P: [Unclear]

I: Rumors about mental disorder person in society means some people treat these patients as if they should be kept in distance as they think these kind of patients are harmful or they humiliate them so in this way there are some kind of rumors around

P: NO these kind of treatments and rumors can be seen in cycosis patient cases because these kind of patients behavior changes time to time as we can't expect their behavior so they can be aggressive anytime but here we don't have any cases like that so I haven't heard about all these rumors around

I: The main problems are created by rumors because of them most of the people don't implement their ideas

P: I never went to field regarding these kind of cases they usually come here but I never went to field

I: So you don't have idea about the these kind of rumors

P: No I don't have idea about these kind of rumors

I: As a doctor what kind of steps must be taken to remove these kind of rumors in public

P: First we have to fill the communication gap we must address the people and if the message is given by doctor they may don't care more but if this message is given by village sarpanch/head it may reach village people more usually in the meeting we must bring any famous personalities he/she may be politican or any person who can draw the people's attention

I: You mean the people who belong to local region must give the message as it may be effective

P: I mean we must also besides him/her I am telling this because the person among them must tell them to think on it then it may be effective and works more

I: Yes some people say that they may give preference to those people who came from outside rather than local person

P: If we tell them they as we are familiar to them they won't be interested so a new person who is outside of village must address the people along with the local person

I: As a doctor what kind of suggestion give for this?

P: About CMD

I: About rumors on CMD's

P: Every person have problems but if he is not able to identify it may get advanced some people feel depressed but when we observe such kind of people we must try to ask him and communicate with him may solve most of the problem, if you are feel emotion depression due a person talk straightly to him/her rather than keeping in you and feel depression that person may also have some problems to behave like that so the solution is communication and sharing

I: The question which I am asking is as there are many rumors about mental disorders and awareness is less so as a doctor what will you do to create awareness in the society?

P: health education and awareness camps may work better to create awareness among public and it is better to address overall rather than subject wise and if it is personal we must ask them to meet as and then we can solve the problem

I: No personally

P: If it is for society then awareness is better option

I: And in awareness camp as you told that we must bring local bodies and doctors so apart from tem do you think the meeting will be successful if we bring film stars or famous politicians?

P: People may come to see the film star but they might not give attention to what he is saying?

I: You mean to say that they ay come to see him not to listen him?

P: If you call pavan kalyan It may be different and if you call Rajeev kanakala then it is a different thing because if those celebrites are famous then they may listen but not sure some people may listen I don't say that they won't but it differs depending on age adult may not give attention as the old age people do

I: So it may work?

P: Yah but I am not sure

I: Do you think there are any kind of rumors about mental disorder in health workers?

P: No I think there are no rumors if the patient behave as psychosis they try to avoid that patient they may hesitate like the people who shout out without any reason or suddenly if those kind of cases exists then they try to ignore especially in government hospitals they afraid of these kind of psychosis because they may get hurt by that person so usually they ignore these kind of cases as it depend on our interest so in private hospital they may take care but depend on person's interest

I: The health worker who work under you have they talk about these kind of mental disorder topic with you anytime?

P: They asked me if we have to ask any question relating to mental disorder especially our asha's asked me this I felt happy that they are thinking to give a start about this topic so I told them to ask causally and not to point out anything just ask them how they feel what are they thinking like that I told them that not to say that we are especially conducting for mental disorder purpose

I: We can also feel happy if they ask any kind of questions to the people it will be helpful to us

P: So when there was meeting with asha's I was not available so they came to me an told that you asked them to question the people about these mental disorder so they asked me whether they can proceed or not so I told them not to use the word "mad" Anywhere I just told them to know there mental status like how are they feeling etc.

I: Thanks you so much in case if there is any rumor in health worker

P: I think if there is any rumor in the mind of health worker they must not come to this field

I: Suppose they don't have awareness' about this

P: When they start doing this survey well they may automatically get some awareness so they have to study then we can give awareness so the question which are asked must be good and must grab more details from them so the way of question must be in good way

I: So according to you how important is it to go for the treatment of common mental disorder?

P: Identification is more important prior to treatment and I cant say treatment but counseling is the best thing means CMD counseling must be made but for SMD's they have to take treatment compulsorily because for CMD people may thing why they should take medication so counseling is good

I: Now you are talking about CMD

P: And if we label any person as SMD then he/she must take medicines counseling it not enough for them so cmd must be given counseling and smd patient with counseling medication is also important

I: Do you think that the treatment for these kind of mental disorder is available easily to people and do you think the people can afford for its treatment?

P: We don't see any kind of symptoms deal with such of kind of patients

I: You mean there is no where

P: There may be one or two in committees but they are specifically label it as mental hospital if a person ids going for treatment he will be labeled as a psychiatric patient so the name matters a lot like for example if a person go into HIV centre and coming out if someone see him there they may doubt on him if he has that disease so due to this many of the people don't go for treatment like many people come here for B.P And sugar checkup there is no problem to the people to come over here but they may feel awkward to go to psychiatrist

I: So due to the name of hospital also can affect the society thinking

P: Yes there are some hospital in Kerala I don't know if we have any kind of hospitals in Andhra

I: Do you think the treatment for mental disorder is affordable?

P: Now if a person come with the complaint of sleepless ness at night we ask them if he have any tensions or anything like that so we usually give some kind of drugs so it is not a problem so due to the drug person may sleep peacefully so by this his way of thinking or any kind or depression may also be reduced

I ; As you told now that we don’t have treatment available near us so question regarding it?

P: Ok

I: So for better treatment and services for mental disorder patient what kind of steps must be taken in your community?

P: First thing is identification In CMD case it can be reversible but not in the case of SMD So for OP we must assign more time to talk to them so it will help to identify any CMD symptoms in person so if we can identify it earlier and give counseling then the problem may reduce

I: So you think that there are no good facilities to these kind of patients so what can of steps must be taken to make facilities available to those kind of patients

P: If a person have mental health problem they afraid to say out the person who deliberately is a mad person so social stigma is showing effect on it because people are afraid to share their situation because they are afraid if they will be labeled ad mad person in the society so we cant keep a board out side for mental health person as I said awareness camp is good as they won’t come here , It is better to maintain stats and let them know about it in awareness camp

I: Do you think to provide some kind of information to your locality or community which can increase the awareness on these kind of mental disorders so if you want to provide such kind of message who will you choose to address people?

P: The person who is fluent in talking it must be interesting and people must show interest in listening him so we must not make long session the session must be short and brief the speech must create awareness in the people or the people who has taken this kind of treatment earlier can convey the message well

I: So mean to say that the person who faced these kind of problem and who is fine after treatment must be chosen to address the people then it may create more awareness in public

P ; Yes We have seen in HIV campaign so first they talk casually to remove awkwardness in public and slowly starts the topic so this kind of strategy may work here too.

I: As we discussed earlier that local bodies, popular persons and the person who came out of that problem after treatment so apart from all these do you think is there any other person who is correct to convey this message volunteers or government

P: I am not sure but how many of people may attend but it is better to choose a person who can talk freely about it

I: You mean volunteers, government, religious persons or teacher

P ; Teacher is the good thing school session is also good mainly for the student above 8 and 9th class ilike upper primary class is good so it is the age which must be moulded as they start to enjoy in different way so it is better to motivate them at that age so it is better to conduct meeting once in a month mainly for girls just like discussion not like class or speech some of the student feel stressed due to studies and all so it is good to take out stress

I: Where must we conduct these kind ofawarness camp at home or any other place where people may come in large volume?

P: panchayt office is good as many people come there

I: Can all of the population can attend this if we keep in panchayat office as we have 5000 population in here for example so can they come from their places to here?Or should we have to divide it in four part and conduct once in one place?

P: Area wise is good idea the local persons must inform them

I: Canvasing must be used

P: Yes but main thing is we can't label it as a mental disorder camp in the word itself they may understand it relating to some mental problems so it may create issues so you have to keep a name like awareness camp that it then only people may come

I: Yes awareness camp

P: [unclear]

I: So you suggest sector wise is the better solution instead of bulk

P: yes it may help to interact and also easy to them to attend

I: Do you want to give any suggestion about this camp, treatment or mobilization etc

P: Patient must be given id rather than name because they may feel awkward to relive their names before

I: We are not putting any name

P: That good thing and other thing is awareness camp which must be held once in a month not frequently

I: They may forget

P: Mental health issue meeting must be frequently held and it may show positive effect as it is sensitive issue we must also be handled sensitively must try to identify and must convenience them for treatment

I: So once in a month is better than best

P: I don’t mean it but if we can share their problem and if we can interact with them then it is best person to person is the better way

I: Thanks sir thank you so much

P: Thanks
